# Structural Origin of the Fast Polymerization Rates and Monomer Universality of Pyrazole-Based Photoiniferters

**DOI:** 10.3390/molecules30183687

**Published:** 2025-09-10

**Authors:** Bo Wang, Xuegang Liu, Zhilei Wang, Chenyu Wu, Zikuan Wang, Wenjian Liu

**Affiliations:** 1 Qingdao Institute for Theoretical and Computational Sciences, Center for Optics Research and Engineering, Shandong University, Qingdao 266237, China; 201912125@mail.sdu.edu.cn (B.W.); liuwj@sdu.edu.cn (W.L.); 2 Center of Basic Molecular Science, Department of Chemistry, Tsinghua University, Beijing 100084, China; lxg23@mails.tsinghua.edu.cn

**Keywords:** RAFT polymerization, photoiniferter, chain transfer agent, controlled polymerization, DFT

## Abstract

Herein, we report a combined computational and experimental investigation into the recently reported universal pyrazole-based reversible addition-fragmentation chain transfer (RAFT) agents (Z−C(=S)−S−R, where Z is 3,5-dimethyl-1*H*-pyrazol-1-yl), which can mediate controlled radical polymerization of a broad scope of monomers without the need for an additional initiator or catalyst. The results reveal that the high molar absorption coefficient and efficient photolysis kinetics of pyrazole-based chain transfer agents (CTAs) under blue light ($\lambda_{\max}$ = 465 nm) enable rapid radical generation, underpinning ultrafast polymerization of acrylates, acrylamides, methacrylates, and *N*-vinylpyrrolidone (NVP). While the efficient light absorption is attributed to structural dissimilarity between the Z group and the S–R group (which breaks the local symmetry of the C=S group), the fast photolysis originates from favorable π electron donation from the Z group to the C=S group. Meanwhile, the π electron donation is still weaker than in xanthates, which explains the excellent control of a wide range of monomers, except methacrylates. This work establishes design principles for next-generation CTAs for ultrafast and monomer-universal photoiniferter RAFT polymerization.

## 1. Introduction

The precise synthesis of polymeric materials with defined sequences, akin to biomacromolecules, is a major goal in polymer chemistry [1,2]. Reversible deactivation radical polymerization [3,4,5,6,7,8,9,10] (RDRP) enables this pursuit by enabling monomer insertion to be paused and restarted via stop–restart cycles, thus homogenizing molecular weights and facilitating control over backbone architecture, chain length, block sequence, and composition [3]. However, achieving sequence definition across diverse monomer classes (acrylates, acrylamides, methacrylates, methacrylamides, vinyl acetates, and vinyl amides) is challenging, as different monomers require specific controlling end groups [11,12]. Developing universal reversible addition-fragmentation chain transfer (RAFT) agents, compatible with broad monomer scopes, is deemed critical to practical block copolymer synthesis [13]. In addition, it is desirable that the polymerization is achieved at a high reaction rate, which is vital to applications like self-healing gel [14] or 3D printing [15,16].

Conventional RAFT agents (hereafter also denoted chain transfer agents, CTAs), which have the general formula Z−C(=S)−S−R, exhibit monomer-specific efficacy: dithioesters [17,18] and trithiocarbonates [19] (where Z is a carbon or sulfur substituent, respectively) excel with more active monomers (MAMs) but give low or negligible polymerization rates for less active monomers (LAMs), while xanthates [20,21] or dithiocarbamates [22,23] (where Z is an oxygen or nitrogen substituent, respectively) favor LAMs but lack control for MAMs. The fundamental reason lies in the conjugative stabilization effects of the Z group: Z groups with strong conjugative electron-donating ability destabilize the chain transfer intermediate (CTI) and are thus more suitable for LAMs, and Z groups without such an ability are accordingly more suitable for MAMs. This dichotomy complicates block copolymer synthesis involving both LAMs and MAMs, necessitating complex strategies like RAFT agent switching [11,24,25,26,27,28,29,30,31] or mechanistic transitions [32], where the structure of the CTA is changed by, e.g., protonation or deprotonation, to accommodate the new monomer.

**Figure 1:**
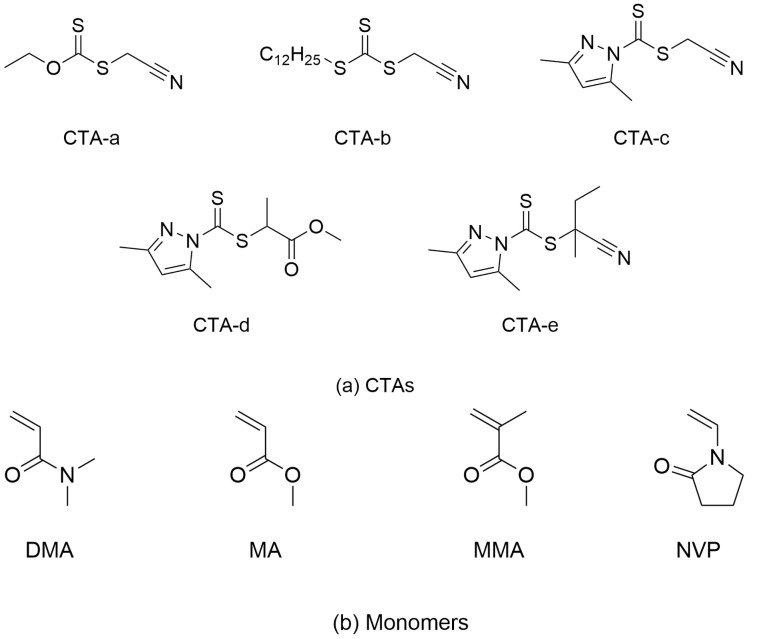
Chemical structures of (**a**) CTAs and (**b**) monomers used for photoiniferter RAFT polymerization.

To obtain the best possible compromise between LAMs and MAMs, one may design CTAs with intermediate conjugative stabilization effects between those of electron-rich (oxygen/nitrogen) and electron-poor (carbon/sulfur) substituents. Fortunately, the easily accessible pyrazole-based CTAs [13,23,33] (e.g., 3,5-dimethyl-1*H*-pyrazol-1-carbodithioates) sit comfortably in such a position: while their Z groups are attached to the −C(=S)−S−R moiety through a nitrogen atom, the nitrogen atom’s lone-pair electrons are delocalized onto the π system of the pyrazole ring (cf. Figure 1a), making such CTAs less electron-rich than typical dithiocarbamates but still more electron-rich than trithiocarbonates. Hence, they enable thermally initiated RAFT polymerization of both MAMs and LAMs with moderate control [23].

The benefits of universal pyrazole-based CTAs in synthesizing complex block copolymers are further amplified in photoiniferter polymerization [13]. In this system, the CTA serves not only as a chain transfer agent but also as a photoinitiator and a terminator [14,34,35]. This multifunctionality eliminates the need for external initiators or terminators, enabling the synthesis of pure, well-defined block copolymers with minimal dead chains from termination events and ensuring high end-group fidelity [35]. Critically, the process offers temporal control by visible light, providing a facile route to synthesizing complex block copolymers where high end-group fidelity and precise temporal control are essential. Indeed, the pyrazole-based CTAs for photoiniferter polymerization reported by Lian et al. were found effective in synthesizing MAM-LAM block copolymers (e.g., poly(*N,N*-dimethylacrylamide) (PDMA)-*b*-poly(*N*-vinylpyrrolidone) (PNVP)), with polymerization rates that are one order of magnitude higher than xanthates and trithiocarbonates, yet compatible MAMs are limited to acrylates and acrylamides [13]. Methacrylates can hardly be incorporated due to suboptimal molecular-weight control [13]. Further optimization of the universality of CTAs to improve the control of methacrylates while not deteriorating the excellent polymerization rate is particularly desirable, necessitating thorough computational analysis because of the mechanistic complexity of photoiniferter systems.

Herein, we present a computational study dissecting the mechanism of pyrazole-based photoiniferter RAFT polymerization. By combining time-dependent density functional theory (TD-DFT), quantum chemical calculations, kinetic simulations, and transient absorption spectroscopy (TAS), we

1. Quantify photophysical properties (absorption spectra, intersystem crossing (ISC) rates, and internal conversion (IC) rates) and photolysis kinetics of pyrazole-CTAs under blue light, rationalizing the efficient light absorption and rapid photolysis of these CTAs;
2. Evaluate chain transfer and fragmentation kinetics for MAMs (e.g., methyl acrylate (MA), *N,N*-dimethylacrylamide (DMA), and methyl methacrylate (MMA)) and LAMs (NVP);
3. Identify why acrylates/acrylamides achieve good molecular-weight control while methacrylates suffer from termination and broad dispersity;
4. Propose molecular design strategies for CTAs with enhanced monomer universality.

This work bridges computational insights with experimental advances, providing a roadmap for next-generation photoiniferters capable of controlling methacrylates at little loss of polymerization rate—a critical step toward truly universal RAFT polymerization.

## 2. Results and Discussion

**Figure 2:**
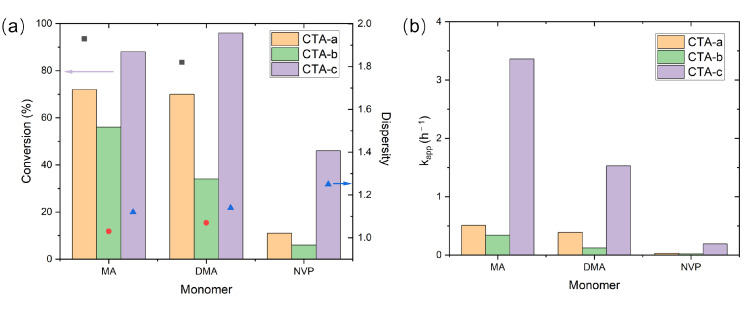
Photoiniferter RAFT polymerization results for different CTAs. (**a**) Monomer conversions (bars) and dispersity indices (symbols); (**b**) apparent polymerization rates ($k_{\mathrm{app}}$). The dispersity indices of NVP with CTAs *a*-*b* are not reported due to low monomer conversion. Data taken from Ref. [13].

The referenced experimental data (Figure 2) for photoiniferter RAFT polymerization were selected from the reported study by Z. An’s group [13]. The CTAs (Figure 1a) studied here include xanthate (CTA-*a*), trithiocarbonate (CTA-*b*), and dithiocarbamates (CTA-*c*∼CTA-*e*), while the monomers (Figure 1b) under investigation include not only MAMs (MA, DMA, and MMA) but also LAMs (NVP).

The experimental results (Figure 2; Table S4) revealed significant variations in polymerization performance among CTAs *a*-*c* with different Z groups when synthesizing poly(MA) (PMA), PDMA, and PNVP. Notably, the pyrazole-based CTA-*c* achieved both the highest monomer conversion and the fastest polymerization kinetics for all three monomers. Meanwhile, for MMA (whose data are not shown in Figure 2, since only CTAs *c*-*e* were tested; see Table S4), the control of polymerization was much inferior to the best achievable control of MA, DMA, and NVP, even with the optimal CTA (CTA-*d*), which gave a dispersity of 1.59 [13]. This suggests that further improvement of the Z group is necessary for controlling the polymerization of MMA without deteriorating the performance of other monomers.

**Figure 3:**
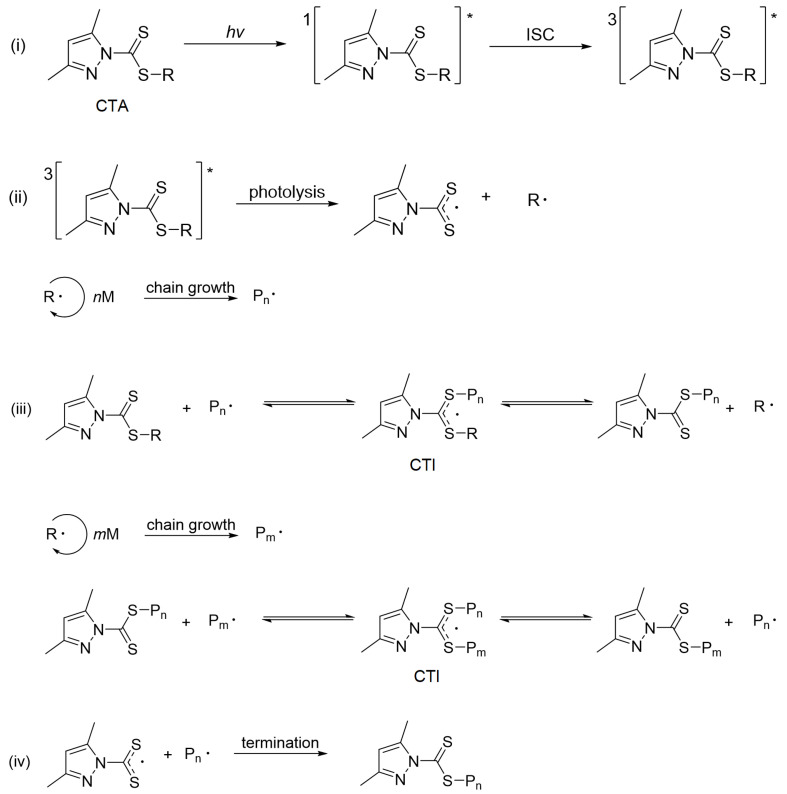
General mechanism for photoiniferter RAFT polymerization using pyrazole-based CTAs ("*" denotes excited states). (**i**) photoexcitation, (**ii**) initiation, (**iii**) chain transfer, and (**iv**) termination.

To rationalize the influence of Z groups on polymerization performance, we conducted detailed theoretical analyses of the photoexcitation, initiation, and chain transfer steps (Figure 3) to explain the fast kinetics of pyrazole CTAs compared with non-pyrazole CTAs (CTA-*a* and CTA-*b*) and their superior control across diverse monomers (MAMs and LAMs). Further, we point out possible future directions for improving the control of MMA polymerization.

### 2.1. Photoexcitation

The photophysical properties of CTAs were investigated first (Table 1). The computed excitation energies (obtained using ORCA [36,37,38,39,40]) agree closely with the experimental data, confirming the reliability of our level of theory. Pyrazole CTAs are predicted to have higher S_1_ absorption oscillator strengths than non-pyrazole CTAs (i.e., CTA-*a* and CTA-*b*), agreeing with the trend of experimental molar absorption coefficients and suggesting that pyrazole CTAs absorb more light than non-pyrazole CTAs, thereby facilitating photolysis (Figure 2).

**Figure 4:**
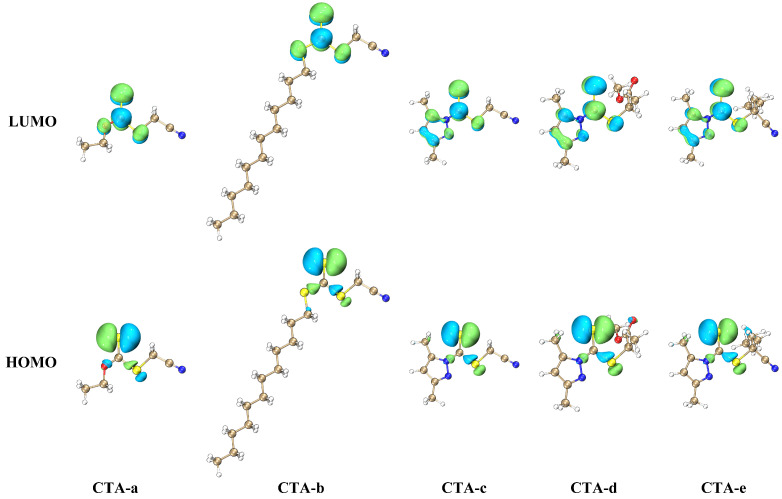
HOMO and LUMO of the CTAs studied in the present work, plotted using VMD [41] with cube files generated by Multiwfn [42].

The high molar absorptivity of pyrazole CTAs can be rationalized based on symmetry arguments. The S_1_ states of CTAs are dominated by the HOMO→LUMO transition (> 95%) and have $n\left(S\right)\to \pi^{*}$(C=S) character (Figure 4). If the CTA is a symmetric trithiocarbonate (RS)_2_C=S, the molecule would have C_2v_ symmetry, so that the $n\left(S\right)\to \pi^{*}\left(C=S\right)$ state belongs to the A_2_ irreducible representation; the S0→S_1_ absorption is, therefore, dipole-forbidden. While CTA-*b* is not a symmetric trithiocarbonate, such that it does not exhibit exact C_2v_ symmetry, the trithiocarbonate group is still characterized by an approximate C_2v_ symmetry, and S0→S_1_ absorption remains almost forbidden ($f\;=\;10^{-6}$). In contrast, the pyrazole Z group significantly disrupts the local C_2v_ symmetry of the C=S group, leading to a drastic increase in the oscillator strength ($f\approx 10^{-4}$) and a less dramatic but still notable increase in the experimental absorption coefficient (Table 1). In particular, the LUMOs of all pyrazole-based CTAs have a node on the nitrogen atom directly bonded to the C=S group, and a lobe on the sulfur atom bonded to the R group; in xanthates and trithicarbonates, lobes are observed on both of the two atoms bonded to the C=S group (Figure 4). We thus conclude that the enhanced polymerization rate of pyrazole-based CTAs can be partly attributed to the broken local symmetry of the C=S group.

**Figure 5:**
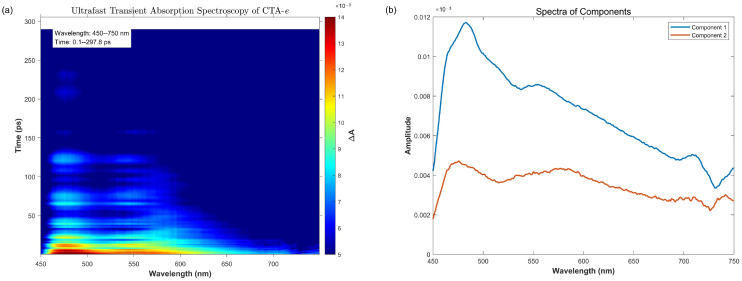
Femtosecond transient absorption spectrum results. (**a**) Ultrafast transient absorption spectroscopy of CTA-*e* (Δ*A* represents the change in absorbance). (**b**) The spectra of different kinetic components extracted from (**a**) (refer to the Supplementary Materials for related code). The fitted time constants for components 1 and 2 are 30.55 ps and 8367.97 ps, respectively.

The next critical mechanistic question concerns which intermediate excited states are involved in the photophysical and photochemical processes. To resolve this, we conducted TAS studies using CTA-*e* as a representative system (Figure 5). The exponential fit of the experimental data reveal two components with exponential decay time constants around 31 ps and 8 ns, respectively, while fitting the data with three components did not result in an appreciable improvement in the fit (Table S1, Supporting Information). Both components are characterized by two transient absorption peaks at 450–500 nm and 550–600 nm, respectively, consistent with the computed transient absorptions of both the S_1_ (481, 525, 637 nm) and T_1_ (442, 576 nm) states. We, therefore, assign the small time constant to the decay of the S_1_($n\left(S\right)\to \pi^{*}\left(C=S\right)$) state and the large time constant to the decay of the T_1_($n\left(S\right)\to \pi^{*}\left(C=S\right)$) state. The absence of a third component suggests that other excited states (e.g., T_2_) do not reach a significant concentration during the excited-state relaxation process.

### 2.2. Initiation

The S_1_ and T_1_ states of CTAs are unstable and prone to C–S bond homolysis to yield a thiocarbonylthio radical (bearing the Z group) and an R radical. The resulting R radical can then serve as an active species to initiate monomer polymerization. Consequently, the photolysis efficiency of the S_1_ and T_1_ states of the CTA directly correlates with the polymerization rate. The present subsection will focus on the photolysis of the T_1_ state, followed by a brief discussion of the photolysis of the S_1_ state.

The photolysis rate is inversely correlated with the Gibbs free energy barrier ($\Delta G_{\mathrm{photolysis}}^{T_{1}}$) for the T_1_ state C–S bond homolysis, via the Eyring equation (Equation (1), where $k_{\mathrm{photolysis}}^{T_{1}}$ is the photolysis reaction rate, $k_{B}$ is the Boltzmann constant, *h* is Planck’s constant, *T* is the temperature, and *R* is the gas constant):

(1) $$k_{\mathrm{photolysis}}^{T_{1}}\;=\;\frac{k_{B}T}{h}\exp\left(-\frac{\Delta G_{\mathrm{photolysis}}^{T_{1}}}{RT}\right)$$

**Figure 6:**
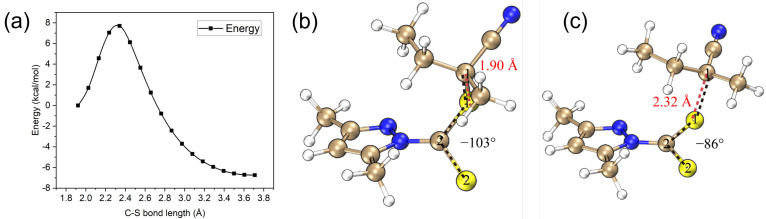
(**a**) Relaxed scan of the C–S bond for the T_1_ state of CTA-*e* at the ωB97X-D3/def2-SVP/SMD-DMSO level. (**b**) The T_1_ equilibrium geometry of CTA-*e*. (**c**) The T_1_ TS geometry of CTA-*e*. The red and black dashed lines in (**b**,**c**) highlight the C-S bond and S_2_-C_2_-S_1_-C_1_ dihedral, respectively.

The photolysis transition state can be conveniently located using a relaxed scan of the C–S bond (e.g., Figure 6a). The C–S bond of the T_1_ equilibrium geometry is elongated from 1.90 *Å* at the equilibrium structure to 2.32 *Å* at TS (Figure 6b,c), suggesting a significant weakening of the C–S bond. Moreover, the dihedral angle of S_2_–C_2_–S_1_–C_1_ changes from -103 to -86, yielding a better spatial alignment of the $\pi^{*}\left(C=S\right)$ and $\sigma^{*}\left(S–R\right)$ orbitals, which is vital to the smooth transition of the T_1_ state from $n\left(C=S\right)\to \pi^{*}\left(C=S\right)$ to $n\left(C=S\right)\to \sigma^{*}\left(S–R\right)$ character and therefore S–R bond cleavage [43].

As shown in Table 1, CTA-*a* and CTA-*b* exhibit comparable $\varepsilon_{\max}$ values; however, the higher $\Delta G_{\mathrm{photolysis}}^{T_{1}}$ of CTA-*b* (Table 2) would correspond to a slower polymerization rate, which aligns with the experimentally observed polymerization rates for MA and DMA (Figure 2b). In contrast, while CTA-*c* possesses a higher $\Delta G_{\mathrm{photolysis}}^{T_{1}}$ than CTA-*a* (Table 2), its elevated $\varepsilon_{\max}$ (as well as the fact that the absorption maximum of CTA-*c* is in the visible region while that of CTA-*a* is in the ultraviolet region) results in a higher polymerization rate (Figure 2b). These findings demonstrate that the polymerization rate of CTAs is governed by both $\varepsilon_{\max}$ and photolysis efficiency. Nevertheless, if we exclude CTA-*a*, the T_1_ photolysis rates of the remaining four CTAs correlate very well with the experimental apparent photolysis rates in the presence of 2,2,6,6-tetramethylpiperidinoxy (TEMPO) as a radical trap (from CTA-*b* to CTA-*e*: 0.004, 0.057, 0.194, and 11.17 h^−1^; see Figure S8 of Ref. [13]).

To rationalize the Z group dependence of the T_1_ photolysis rates, we compared the T_1_ photolysis rates of CTAs *a*-*c* with the C=S bond lengths of the T_1_ state, revealing a positive correlation (Table 2). A longer C=S bond length signifies stronger conjugative electron donation from the Z group to the C=S bond. The present data, therefore, support the view that conjugative electron donation of the Z group destabilizes the T_1_ equilibrium geometry relative to the T_1_ TS (which has more double-bond character than the T_1_ equilibrium geometry and thereby forms stronger conjugative interactions with the Z group) and thus reduces the photolysis barrier. We also calculated the atomic dipole moment corrected Hirshfeld (ADCH) charges of the Z groups and the CS_2_ units of the S_0_ and T_1_ states of the CTAs (Tables S2 and S3) but did not find any monotonic correlation with the T_1_ photolysis rate. One possible reason is that the atomic charges contain contributions from both inductive and conjugative effects.

Similar to the T_1_ state, the S_1_ state can also undergo S–R bond photolysis, owing to its similar excited-state character ($n\left(S\right)\to \pi^{*}\left(C=S\right)$) to the T_1_ state. Due to spin contamination of the S_0_ reference state in the dissociation region, the S_1_ photolysis rate of CTA-*e* was calculated using the spin-flip Tamm–Dancoff approximation (SF-TDA) [44] at the aforementioned level of theory, yielding a value of 4.2 × 10^7^s^−1^, which matches the T_1_ photolysis rate (5.3 × 10^7^s^−1^) within computational error.

**Figure 7:**
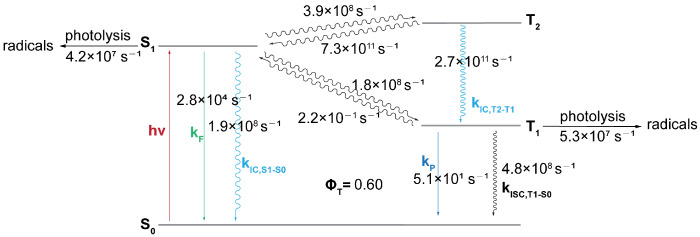
Schematic representation of the Jablonski diagram of CTA-*e*, with computed rate constants (F: fluorescence; P: phosphorescence; IC: internal conversion; ISC: intersystem crossing).

**Figure 8:**
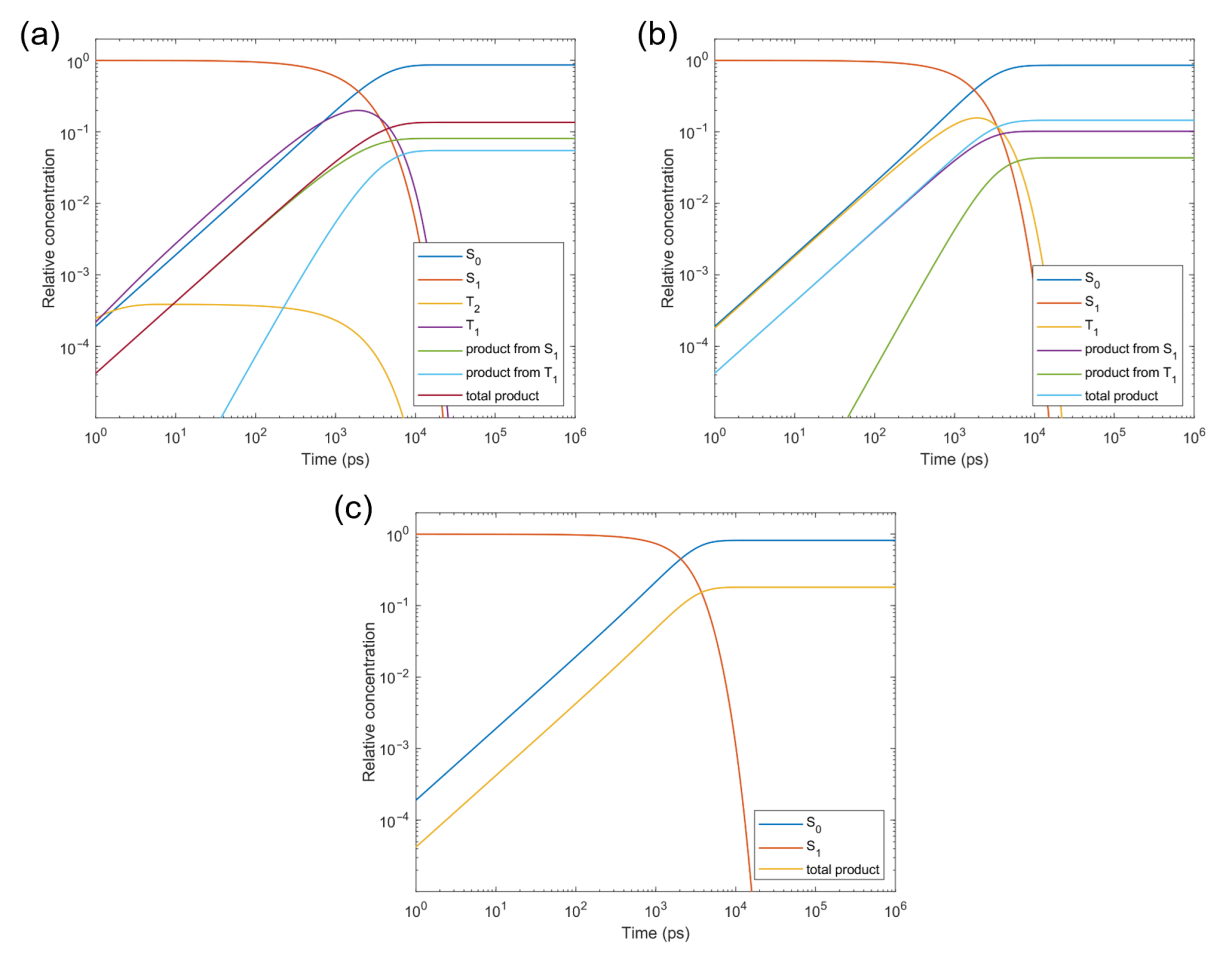
Kinetic simulations of the excited-state relaxation of CTA-*e*, starting from the S_1_ state (**a**) with all pathways taken into account (photolysis quantum yield: 13.6%), (**b**) with all pathways involving the T_2_ state removed (photolysis quantum yield: 14.5%), and (**c**) with all pathways involving the T_1_ and T_2_ states removed (photolysis quantum yield: 18.1%).

To elucidate the relative importance of the S_1_ and T_1_ photolysis pathways, we calculated the transition rates between the S_1_, T_2_, T_1_, and S_0_ states of CTA-*e* (Figure 7) and performed kinetic simulations based on the computed rates (Figure 8). The S_1_→T_2_ ISC is El-Sayed-allowed [45] (spin–orbit-coupling matrix element (SOCME): 127 cm^−1^ due to the change in excited-state character from ($n\to \pi^{*}$) to ($\pi \to \pi^{*}$). However, as this ISC process is endothermic, it is accompanied by an even faster T_2_→S_1_ reverse ISC (RISC) process, which results in a negligible steady-state concentration of the T_2_ state (3.9 × 10^−4^ relative to the initial S_1_ concentration; Figure 8a) and is consistent with our inability to observe the T_2_ state in the TAS results (Figure 5). Although the T_2_ state can quickly relax to the T_1_ state with a rate of 2.7 × 10^11^s^−1^ (Figure 7; nonadiabatic coupling matrix elements [46] used for computing the T_2_→T_1_ rate were obtained using BDF [47]), it does not outcompete the T_2_→S_1_ RISC. Consequently, the photolysis quantum yield is almost unaffected when we remove all relaxation pathways involving T_2_ (Figure 8b), suggesting that the T_2_ state is not an important intermediate state. S_1_→T_1_ ISC is El-Sayed-forbidden ($\left(n\to \pi^{*}\right)\to (n\to \pi^{*}$), SOCME: 5 cm^−1^) and effectively irreversible owing to a large S_1_-T_1_ gap; the fact that the T_1_ adiabatic excitation energy (1.70 eV) is much lower than the S_1_ adiabatic excitation energy (2.16 eV) also results in a faster T1→S_0_ ISC rate than the S1→S_0_ IC rate, despite the spin-forbidden character of the former. Therefore, although T_1_ photolysis makes a similar contribution (5.5%) to the total photolysis quantum yield (13.6%) compared with S_1_ photolysis (8.1%), the total photolysis quantum yield increases to 18.1% when all relaxation pathways involving T_1_ and T_2_ are removed (Figure 8c). These results highlight the seemingly contradictory roles of the triplet states of pyrazole-based CTAs, which yield a significant portion of the photolysis products but, in the meantime, make a negative contribution to their quantum yield, since the contribution of T_1_→S_0_ ISC is greater than the contribution of T_1_ photolysis. Finally, we note that the T_1_ concentration is predicted by the kinetic simulations (Figure 5a) to rise on the timescale of a few hundreds of picoseconds, and fall on the timescale of a few nanoseconds, in qualitative agreement with our TAS data (Figure 5).

### 2.3. Chain Transfer

While the photolysis quantum yield correlates with the polymerization rate, the controllability of the polymerization is intimately related to the efficiency of chain transfer, where a chain-propagating radical reversibly adds to the C=S group of the CTA. As the steady-state concentration of the propagating radical is low, the addition rate constant of the P_n_ radical to the C=S bond ($k_{\mathrm{CT}}^{\mathrm{II}}$) must be sufficiently large for efficient chain transfer. The ratio of $k_{\mathrm{CT}}^{\mathrm{II}}$ to the S-P_n_ bond fragmentation rate constant of the chain transfer intermediate (CTI) ($k_{\mathrm{CT}}^{I}$) determines the steady-state ratio of CTI to propagating radical; a small $k_{\mathrm{CT}}^{\mathrm{II}}/k_{\mathrm{CT}}^{I}$ ratio yields a high propagating radical concentration and thus faster polymerization.

The computed chain transfer rate constants are shown in Table 3. As expected, MMA gives the lowest $k_{\mathrm{CT}}^{\mathrm{II}}$ among all four monomers (MA, DMA, MMA, and NVP), suggesting inefficient chain transfer, which aligns with the poor control observed experimentally. The small $k_{\mathrm{CT}}^{\mathrm{II}}/k_{\mathrm{CT}}^{I}$ for MMA points to a fast polymerization rate, consistent with the experimental observation that the optimal input light intensity for MMA ( 5.0 mW/cm^2^) is lower than for other monomers ( 17.0 mW/cm^2^) [13].

To elucidate the deeper reason of the difference between the chain transfer activities of the monomers, we calculated the structural reorganization energies λ (Table 3), defined as

(2) $$\lambda \;=\;E\left(\mathrm{CTA},\mathrm{frozen}\right)+E\left(P_{n},\mathrm{frozen}\right)-E\left(\mathrm{CTA},\mathrm{optimized}\right)-E\left(P_{n},\mathrm{optimized}\right)$$

where the “frozen” geometries of the CTA and chain-propagating radical P_n_ are taken from the optimized geometries of the respective CTI. Upon the addition of the P_n_ radical to the CTA, the terminal carbon atom of the propagating chain changes from sp^2^ hybridization to sp^3^ hybridization, building up steric strain around the carbon atom. As MMA gives the largest λ among the four monomers, the instability of the CTI of MMA can be explained by steric factors.

Overall, the high $k_{\mathrm{CT}}^{\mathrm{II}}$ and uniform $k_{\mathrm{CT}}^{\mathrm{II}}/k_{\mathrm{CT}}^{I}$ values make pyrazole-based CTAs versatile photoiniferters for acrylates, acrylamides, and vinyl amides, while the low $k_{\mathrm{CT}}^{\mathrm{II}}$ values are primarily responsible for their poor control of methacrylates.

## 3. Discussion

We have conducted detailed analyses on the light absorption, photoinitiation, and chain transfer steps of photoiniferter RAFT polymerization mediated by a few selected CTAs. Our results reveal that pyrazole-based Z groups are advantageous over traditional Z groups (such as alkoxy and alkylthio groups) for the following reasons:

1. The pyrazolyl group is chemically very different from the S–R group in the CTA, which helps to break the approximate C_2v_ symmetry around the C=S group and enhance light absorption.
2. The pyrazolyl group is a sufficiently strong conjugative electron-donating group, giving lower excited-state S–R bond dissociation barriers than trithiocarbonates, which increases the apparent polymerization rate.
3. Meanwhile, the electron-donating ability is not overly strong, enabling the C=S bond to readily accept attacks from PMA and PDMA propagating radicals and efficiently mediate chain transfer—unlike xanthates, which underperform in controlling MA and DMA polymerization. The addition of PNVP propagating radicals to CTA-*e* is faster than for PMA and PDMA, but its dissociation from the CTI is also accelerated, yielding reasonably fast NVP polymerization (though still one order of magnitude slower than MA/DMA).

An interesting question is whether pyrazole or similar groups are essential to fast visible-light polymerization and monomer universality. Points (2) and (3) suggest that the optimum Z group should have intermediate π electron-donating ability between alkoxy and alkylthio groups. Considering that dialkylamino groups are more π-donating than alkoxy groups, while alkyl/aryl groups are less π-donating than alkylthio groups, four strategies can be proposed for universal RAFT iniferters (limiting Z to N, O, S, or C substituents):

(a) Use dithiocarbamates with electron-poor nitrogen substituents;
(b) Use xanthates with electron-poor oxygen substituents;
(c) Use trithiocarbonates with electron-rich sulfur substituents;
(d) Use dithioesters with electron-rich carbon substituents.

Strategies (b) and (c) yield CTAs with approximate C_2v_ symmetry and thus tend to lead to small absorption coefficients. Strategies (a) and (d) produce asymmetrical CTAs with higher absorption. Since dithiocarbamates suit LAMs while dithioesters suit methacrylates, we expect that strategy (a) would be suitable for LAMs, acrylates, and acrylamides but not methacrylates (as observed for 3,5-dimethyl-1*H*-pyrazol-1-yl), while strategy (d) would be suitable for most MAMs but not LAMs.

Incorporating the nitrogen atom of dithiocarbamates into the pyrazole motif reduces electron density through lone-pair delocalization, fitting strategy (a). Indeed, the calculated ADCH charge of the carbamate nitrogen atom of CTA-*e* amounts to +0.107, while changing the 3,5-dimethyl-1*H*-pyrazol-1-yl group to Et_2_N yields a charge of −0.072. Not all conjugated systems perform equally in delocalizing the lone pair, though: pyrrole substituents suit only MAMs, while five-membered lactams favor LAMs over MAMs (likely even worse for methacrylates) [22]. Thus, pyrazole-based CTAs are probably already among the most universal CTAs with comparable polymerization rates and structural simplicity.

Future improvements to methacrylate polymerization could involve:

- Reducing the polymerization temperature, increasing the steady-state concentration of the CTI taking advantage of the exothermicity of CTI formation (Table S5);
- Exploring alternative nitrogen heterocycles (e.g., imidazoles [48] or cyclic imines [49,50]) for finer electronic tuning;
- Adding electron-withdrawing substituents (e.g., chlorine) [51] to pyrazole rings (however, this may slow down LAM polymerization);
- Prioritizing strategy (d) for MMA control (leveraging dithiobenzoates’ excellent performance for MMA polymerization [43]), though highly electron-donating substituents would be needed for LAM compatibility.

To evaluate the latter two approaches, we computed the chain transfer rate constants of the 4-chloro-3,5-dimethyl-1*H*-pyrazol-1-yl- and 4-dimethylaminophenyl-substituted CTAs (Table S6) and found that both exhibit larger $k_{\mathrm{CT}}^{\mathrm{II}}/k_{\mathrm{CT}}^{I}$ ratios (0.74 and 0.46, respectively) compared with the 3,5-dimethyl-1*H*-pyrazol-1-yl-substituted CTAs studied herein (Table 3), suggesting higher chain transfer efficiency. Whether these CTAs can indeed mediate fast and well-controlled polymerization of MMA without deteriorating the performance of other monomers can, however, only be settled by experimental verifications, which are beyond the scope of the present study.

## 4. Conclusions

In summary, we have performed extensive computational and experimental studies on the photophysics, photochemistry, and chain transfer reactions of pyrazole-based RAFT photoiniferters, comparing them with traditional agents (xanthates and trithiocarbonates). Our results rationalize the superior performance of pyrazole CTAs in photoiniferter RAFT polymerization, achieving both high polymerization rates and broad monomer scope. We hope this work inspires development of truly universal RAFT photoiniferters that improve MMA control while maintaining favorable polymerization rates for other monomers, especially LAMs.

## Figures and Tables

**Table 1:** Comparison of experimental [13] and calculated photophysical properties for the longest wavelength absorptions (S_1_ states) of CTAs.

CTA	Exp. $\lambda_{\max}$ (nm (eV))	Exp. $\varepsilon_{\max}$ (L mol^−1^ cm^−1^)	Cal. $\lambda_{\max}$ (nm (eV))	Cal. *f* (10^−4^)	ΔE (eV)
*a*	365 (3.40)	51.0	334 (3.71)	0.7	0.32
*b*	435 (2.85)	38.9	399 (3.11)	0.0	0.26
*c*	420 (2.95)	71.3	385 (3.22)	0.7	0.27
*d*	420 (2.95)	71.6	391 (3.17)	2.3	0.22
*e*	435 (2.85)	66.1	403 (3.08)	1.6	0.23
MAD					0.26

Note: $\lambda_{\max}$, maximum absorption wavelength in DMSO; $\varepsilon_{\max}$, molar extinction coefficient in DMSO; *f*, oscillator strength; ΔE, absolute error of the calculated excitation energy compared with experiment; MAD, mean absolute deviation. Calculation method for vertical absorption of CTAs: TD-DFT/ωB97X-D3/def2-SVP/SMD-DMSO at the S_0_ structures.

**Table 2:** T_1_ state photolysis barriers and rates, as well as T_1_ state C=S bond lengths of the CTAs.

CTAs	$\Delta G_{\mathrm{photolysis}}^{T_{1}}$ (kcal/mol)	$k_{\mathrm{photolysis}}^{T_{1}}$ (s^−1^)	R(C=S) (T_1_, Å)
*a*	5.3	$8.2\times 10^{8}$	1.732
*b*	13.8	$5.1\times 10^{2}$	1.702
*c*	11.4	$2.6\times 10^{4}$	1.711
*d*	10.9	$5.8\times 10^{4}$	1.713
*e*	6.9	$5.3\times 10^{7}$	1.711

Note: Calculations were performed at the ωB97X-D3/def2-SVP/SMD-DMSO level. The photolysis reaction rate constant is calculated from transition state theory.

**Table 3:** Chain transfer properties of the pyrazole-based CTAs with different monomers.

CTA	$\Delta G_{\mathrm{CT}}^{I}$	$k_{\mathrm{CT}}^{I}$	$\Delta G_{\mathrm{CT}}^{\mathrm{II}}$	$k_{\mathrm{CT}}^{\mathrm{II}}$	$k_{\mathrm{CT}}^{\mathrm{II}}/k_{\mathrm{CT}}^{I}$	λ	$M_{n,\mathrm{th}}$	$M_{n}$	*Ð*
	kcal/mol	s^−1^	**kcal/mol**	**L/(mol** · **s)**		**kcal/mol**	**kg/mol**	**kg/mol**	
CTA-MA	15.6	$2.1\times 10^{1}$	14.0	$3.4\times 10^{2}$	16	2.1	38.1	31.4	1.12
CTA-DMA	15.3	$3.7\times 10^{1}$	11.8	$1.4\times 10^{4}$	378	2.1	47.8	49.8	1.14
CTA-MMA	12.5	$4.4\times 10^{3}$	14.5	$1.5\times 10^{2}$	0.034	2.8	48.8	126.4	1.96
CTA-NVP	13.2	$1.2\times 10^{3}$	9.3	$9.4\times 10^{5}$	783	2.2	25.8	8.83	1.25

Notes: Calculations were performed at the ωB97X-D3/def2-SVP/SMD-DMSO level. The CTAs have 3,5-dimethyl-1*H*-pyrazol-1-yl groups as the Z group, and hydrogen-terminated chains containing a single monomer as the R group; for example, CTA-MA is identical to CTA-*d*. The chain-propagating radical undergoing addition to the CTA is likewise hydrogen-terminated and consists of a single monomer. Quantities labeled “I” denote the activation Gibbs free energies and rate constants of CTI fragmentation, while quantities labeled “II” denote those of CTI formation. The chain transfer reaction rate constants were calculated from transition state theory. Experimental data [13] were taken as those of CTA-*c*. Photoiniferter RAFT polymerization conditions: target degree of polymerization (DP) = 500, [MA] = 4 M, DMSO as solvent, blue LED light (465 nm, 17.0 mWcm^−2^), and 30 min Ar deoxygenation. Monomer conversion determined by ^1^H NMR spectroscopy in DMSO-d_6_. $M_{n,\mathrm{th}}\;=\;M_{\mathrm{CTA}}+\mathrm{target}\;\mathrm{DP}\times \mathrm{conversion}\%\times M_{\mathrm{monomer}}$. Molecular weight and dispersity determined by DMF gel permeation chromatography (GPC) equipped with refractive index and light scattering detectors.

## Data Availability

The original contributions presented in this study are included in the article/Supplementary Materials. Further inquiries can be directed to the corresponding authors.

## Supplementary Materials

The following supplementary materials can be downloaded at: https://www.mdpi.com/article/10.3390/molecules30183687/s1. Figure S1. Comparison of transient spectroscopy fitting results using 1∼3 exponentials; Figure S2. Structures of CTA-MMA, CTA-*f* and CTA-*g*; Table S1. Comparison of transient spectroscopy fitting results using 1∼3 exponentials (“cost” represents the sum of squared errors of the exponential fitting model); Table S2. The ADCH charges of the CS_2_ units, R groups and Z groups of the S_0_ states of different CTAs; Table S3. The ADCH charges of the CS_2_ units, R groups and Z groups of the T_1_ states of different CTAs; Table S4. The photoiniferter RAFT polymerization results for different CTAs; Table S5. Activation enthalpies and entropy contributions, as well as reaction enthalpies and entropy contributions of the chain transfer processes of different monomers with pyrazole-based CTAs; Table S6. Chain transfer properties of CTA-*f* and CTA-*g* with MMA. The values of CTA-MMA (Table 3) are shown for comparison. For the meanings of different symbols, please refer to the footnote of Table 3. References [52,53,54,55,56] are cited in the Supplementary Materials.

## Abbreviations

The following abbreviations are used in this manuscript:

CTA	Chain transfer agent
CTI	Chain transfer intermediate
DMA	*N*,*N*-Dimethylacrylamide
DMF	*N*,*N*-Dimethylformide
DMSO	Dimethyl sulfoxide
DP	Degree of polymerization
IC	Internal conversion
ISC	Intersystem crossing
LAM	Less active monomer
MA	Methyl acrylate
MAM	More active monomer
MMA	Methyl methacrylate
NVP	*N*-Vinylpyrrolidone
RAFT	Reversible addition-fragmentation chain transfer
RDRP	Reversible deactivation radical polymerization
SOCME	Spin–orbit-coupling matrix element
TAS	Transient absorption spectroscopy
TEMPO	2,2,6,6-tetramethylpiperidinoxy
