# Supplementary material for: Structural Origin of the Fast Polymerization Rates and Monomer Universality of Pyrazole-Based Photoiniferters
Source: Molecules. 2025 Sep 10;30(18):3687. doi: 10.3390/molecules30183687 (PMC12472791; doi:10.3390/molecules30183687)
Supplement: Supplementary file 1 [file molecules-30-03687-s001.zip › molecules-3855598-supplementary.pdf]

Supplementary Materials
Structural Origin of the Fast Polymerization Rates and Monomer Universality of Pyrazole-Based Photoiniferters

Bo Wang[1], Xuegang Liu[2], Zhilei Wang[1,*], Chenyu Wu[1,*], Zikuan Wang[1,*], Wenjian Liu[1]

[1]Qingdao Institute for Theoretical and Computational Sciences,

Center for Optics Research and Engineering,

Shandong University, Qingdao, Shandong 266237, P. R. China

[2] Center of Basic Molecular Science, Department of Chemistry,

Tsinghua University, Beijing 100084, P. R. China

1. Calculation details

In this work, except for the NACME[1] (non-adiabatic coupling matrix element) and X-TDDFT[2] calculations (performed using BDF[3]), and the thermal vibrational correlation function (TVCF)-based $T_2 \rightarrow T_1$ internal conversion (IC) rate calculations (carried out with MOMAP[4]), all other computations were completed using ORCA[5-9].

The geometry optimization of the $S_0/S_1/T_1/T_2$ equilibrium geometries and T_1 transition states of chain transfer agents (CTAs), as well as chain transfer intermediates (CTIs) and associated transition states was conducted using the ωB97X-D3[10] functional, def2-SVP[11] basis set, defGrid3 integration grid, and SMD[12] solvent model (with DMSO as solvent). The vertical excitation energies of CTAs at the S_0 equilibrium structures were calculated using TD-DFT at the ωB97X-D3/def2-SVP/SMD-DMSO level of theory.

The geometry optimization of the S_1 transition state was performed using the spin-flip TDA[13] (SF-TDA) method at the same theoretical level as specified above. To ensure the consistency of the level of theory, we also re-optimized the S_1 equilibrium structure at this level, such that the energies of the S_1 TS and S_1 equilibrium structure can be subtracted to yield the activation energy. The spin-orbit coupling matrix elements (SOCME) calculations were performed using ORCA, and the NACMEs were calculated using BDF at the same theoretical level as specified above.

All rate constants were computed with the ESD module of ORCA under the harmonic approximation. Due to the limitations of harmonic models in capturing potential energy surface curvature, rate constants for processes involving significant anharmonic effects were calculated using transition state theory (TST), which includes the rates of $S_1 \rightarrow T_2$ and $T_2 \rightarrow T_1$. Specifically, the energy difference of the minimum energy crossing point (MECP) or the minimum energy conical intersection (MECI) with the equilibrium geometry was plugged into the Eyring equation to yield the transition rate. The $T_2 \rightarrow T_1$ internal conversion (IC) rate (2.7×10^{11} s^{-1}) was also recomputed using the TVCF method of the MOMAP software, giving 4.6×10^{11} s^{-1}; their agreement lends further credence to the validity of the results. All rate constant calculations were performed at the same theoretical level as described above.

Finally, at the same computational level described above, we performed calculations of the S_1 and T_1 transient absorption spectra using TDDFT and X-TDDFT, with S_0 and T_1 as the reference states, respectively.

2. Femtosecond transient spectrum of PyZ (CTA-e)

For measurements, the 800 nm fundamental pulses were generated from a Ti: Sapphire laser system (35 fs, 7 mJ/pulse, 1 kHz repetition rate). A 400 nm pulse was used as the actinic pump, which was generated by the second (2ω) harmonic of Ti: Sapphire.

Femtosecond transient absorption (TA) spectra were collected using a commercial femtosecond transient absorption spectrometer (Coherent Astrella + DLChuangRui TA100). A broadband supercontinuum white light with a wavelength range from 350 nm ~ 850 nm serves as the probe pulse by focusing the fundamental beam into a CaF_2 crystal plate, and the full width at half maximum (FWHM) of the instrument response function (IRF) for the entire system is approximately 46 fs. Femtosecond-resolved TA spectra were recorded under 400 nm excitation with power intensity of 3 mW (3 μJ per pulse). Pump power was measured with an optical power sensor (Coherent).

Samples were dissolved in DMSO in an argon-filled glovebox in a 1 mm air-free cuvette, with a concentration (0.2 mol/L) that gave an absorbance of 0.6 at the center of the pump wavelength. Before each measurement, the solution was filtered by a 0.22 μm syringe filter to avoid the influence of scattering on the signal.

Ultrafast spectroscopic results were obtained from the average of three replicate measurements performed under identical conditions. To fix the wavelength-dependent shift in time-zero (caused by varying probe light speeds), chirp correction was applied prior to ultrafast spectroscopic data processing.

Table S1. Comparison of transient spectroscopy fitting results using 1~3 exponentials. ("cost" represents the sum of squared errors of the exponential fitting model)

Num_component	1	2	3
cost	0.321594	0.092474	0.064063
time constant1	200.26 ps	30.55 ps	0.73 ps
time constant2		8367.97 ps	45.70 ps
time constant3			12056.24 ps

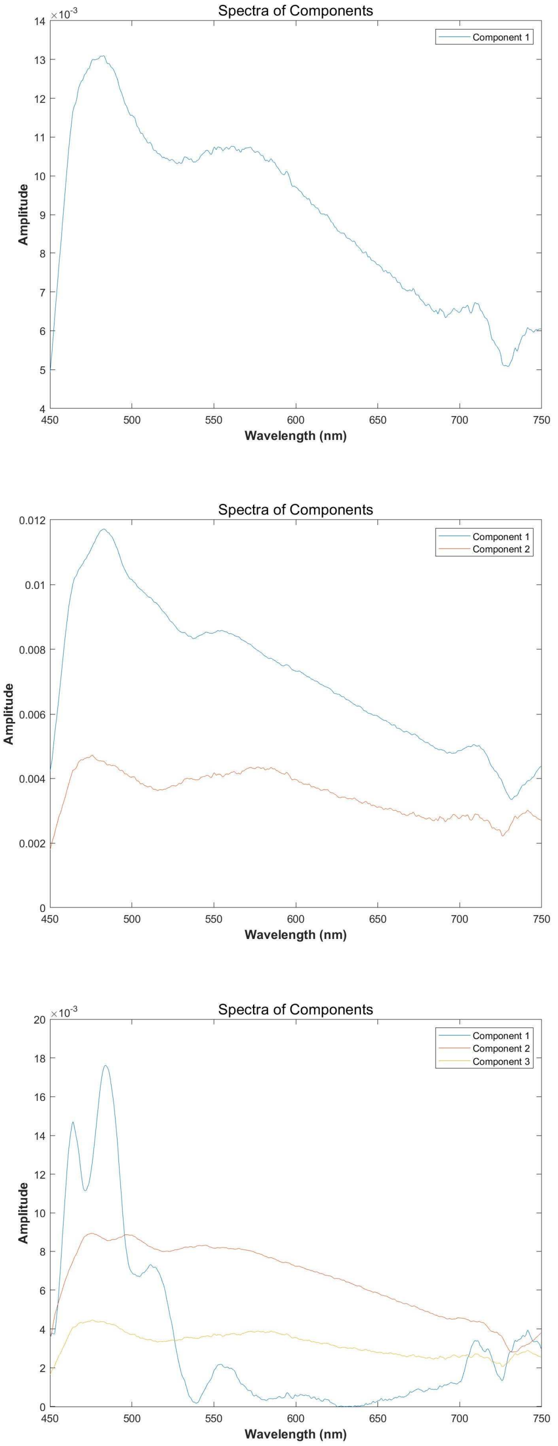

Figure S1. Comparison of transient spectroscopy fitting results using 1~3 exponentials.

From Table S1, we conclude that fitting the transient spectra using two exponentially decaying components gives much lower fitting errors than using a single decaying component. Although there is some further reduction in the fitting error when adding a third exponentially decaying component, the time constant of the added component is very small (0.7 ps), and more importantly, displays large-amplitude oscillations in the 450-600 nm range (Figure S1). It is unclear whether this signal is due to instrument artifacts or e.g. the vibrationally hot S_1 state. It is however relatively certain that this signal is not due to the vibrationally relaxed S_1 state, since the T_1 state is characterized by a broad and featureless spectrum, and the S_1 state, which has a similar composition (n(C=S)→π*(C=S)), is expected to behave similarly. Assuming the three peaks within 450- 550 nm form a vibrational progression, they would correspond to a vibration with a frequency of ~1000 cm^{-1}.

Script for deconvoluting the TAS data into a few exponentially decaying components. The chirp-corrected data files are named Average-chirp-*.csv. The purpose of the following script is to separate kinetic components at different time scales by global multi-exponential fitting. The scripts are given below (note that the user is expected to execute “fs_processing” in the MATLAB command window, while “exponential_fit_cost.m” is an auxiliary script that should be placed in the same folder as fs_processing.m, but should not be executed directly by the user).

fs_processing.m

```
% Read data from file
data1 = readmatrix('C:\Users\user\Desktop\PYZ_FS\Average-chirp-1.csv');
data2 = readmatrix('C:\Users\user\Desktop\PYZ_FS\Average-chirp-2.csv');
data3 = readmatrix('C:\Users\user\Desktop\PYZ_FS\Average-chirp-3.csv');
data = (data1+data2+data3)/3;

% The first row, excluding the first element, is the time (unit: ps)
t = data(1, 2:end);

% The first column, excluding the first element, is the wavelength (unit: % nm)
lam = data(2:end, 1);

% Remove the first row and the first column; this gives the actual data
% Remember the first index is wavelength, and the second index is time
data = data(2:end, 2:end);

% Remove negative peaks due to the laser
lam_laser = 400; % laser wavelength (nm)
dlam = 50;
% only keep wavelength range between lam_laser+dlam and 2*lam_laser-dlam
% Here we use the fact that the second laser peak is at 2*lam_laser
data = data(lam>lam_laser+dlam & lam<2*lam_laser-dlam,:);
lam = lam(lam>lam_laser+dlam & lam<2*lam_laser-dlam);

% Remove data before the pump pulse
data = data(:,t>0);
t = t(t>0);

% Exponential fit: approximate the spectrum data as a sum of a few exponentially decaying species
% Note that we constrain the decay exponents at different wavelengths to be identical
```

```
% initial guess of exponents
exponents = [0.1, 0.01];
exponents = fminunc(@(x)exponential_fit_cost(t,data,x),exponents);

% get the spectra of the components
[cost,y,data0] = exponential_fit_cost(t, data, exponents);

% get the residual
resid = data-data0;

% print optimized exponents, in terms of time constants
Nexp = numel(exponents);
fprintf("cost: %f\n",cost)
for i=1:Nexp
    fprintf("Time constant %d: %.2f ps\n",i,1/exponents(i))
end

% plot the spectra
plot(lam,y(:,1),lam,y(:,2))
legend('Component 1','Component 2')

xlabel('Wavelength (nm)');
ylabel('Amplitude');
title('Spectra of Components');
```

exponential_fit_cost.m

```
function [cost,y,data0] = exponential_fit_cost(t, data, exponents)
% Cost function for the exponential fit.

% Given the exponents, find the fitted spectra using a linear fit
Nexp = numel(exponents);
x = zeros(numel(t),Nexp);
for i=1:Nexp
    x(:,i) = exp(-exponents(i)*t);
end
y = data/x';
% now y(:,i) is the spectrum of the i-th component

% fitted summed spectrum
data0 = y*x';

% Calculate cost (mean square error of the fit)
cost = sum(sum((data - data0).^2));
```

3. Kinetic simulations

To elucidate the relative importance of the S_1 and T_1 photolysis pathways, we calculated the transition rates between the S_1, T_2, T_1 and S_0 states of CTA-e, and performed kinetic simulations based on the computed rates. The script is given below (the user is expected to execute "kinetic" in the MATLAB command window after entering the rate constants).

kinetic.m

```
% Rate constants in s^(-1)
kISCS1T2 = 3.90E+08;
kRISCT2S1 = 7.30E+11;
kICT2T1 = 2.70E+11;
kISCS1T1 = 1.80E+08;
kRISCT1S1 = 2.20E-01;
kF = 2.80E+04;
kICS1S0 = 1.90E+08;
kISCT1S0 = 4.80E+08;
klysisS1 = 4.20E+07;
klysisT1 = 5.30E+07;
kP = 5.10E+01;

N = 7;
k = zeros(N); % S0, S1, T1, T2, dissoc(S1), dissoc(T1), S0(from T1)
% k(final state, initial state) = rate
k(1,2) = kICS1S0+kF;
k(2,3) = kRISCT1S1;
k(2,4) = kRISCT2S1;
k(3,2) = kISCS1T1;
k(3,4) = kICT2T1;
k(4,2) = kISCS1T2;
k(5,2) = klysisS1;
k(6,3) = klysisT1;
k(7,3) = kISCT1S0+kP;

% set diagonal elements of k
for i=1:N
    k(i,i) = -sum(k(:,i));
end

% uncomment this to turn off the T2 pathway
%k(4,:)=0;k(:,4)=0;
% uncomment this to turn off the T1 pathway
%k(3,:)=0;k(:,3)=0;

% simulation
t = 10.^(-12:0.01:-6);
Nt = numel(t);
```

```
c = [0 1 0 0 0 0 0]'; % initial state - 100% S1
c_all = zeros(N,Nt);
for i=1:Nt
    c_all(:,i) = expm(k*t(i))*c;
    % normalize to remove numerical error
    c_all(:,i) = c_all(:,i)/sum(c_all(:,i));
end

% total product yield
tinf = 1;
c_inf = expm(k*tinf)*c;
% normalize to remove numerical error
c_inf = c_inf/sum(c_inf);

% plot

pl = loglog(1e12*t,c_all(1,:)+c_all(7,:),1e12*t,c_all(2,:),1e12*t,c_all(4,:), ...
    1e12*t,c_all(3,:),1e12*t,c_all(5,:),...
    1e12*t,c_all(6,:),1e12*t,c_all(5,:)+c_all(6,:));
legend('S_0','S_1','T_2','T_1','product from S_1','product from T_1','total product',...
    'Location','southeast')

%{
% for the plot without the T2 pathway, use this code instead
pl = loglog(1e12*t,c_all(1,:)+c_all(7,:),1e12*t,c_all(2,:), ...
    1e12*t,c_all(3,:),1e12*t,c_all(5,:),...
    1e12*t,c_all(6,:),1e12*t,c_all(5,:)+c_all(6,:));
legend('S_0','S_1','T_1','product from S_1','product from T_1','total product',...
    'Location','southeast')
% for the plot without the T1 and T2 pathways, use this code instead
pl = loglog(1e12*t,c_all(1,:)+c_all(7,:),1e12*t,c_all(2,:), ...
    1e12*t,c_all(5,:)+c_all(6,:));
legend('S_0','S_1','total product',...
    'Location','southeast')
%}
axis([1e12*min(t) 1e12*max(t) 1e-5 2])
xlabel('Time (ps)')
ylabel('Relative concentration')
set(pl,'LineWidth',1)

yield = c_inf(5)+c_inf(6);
text(1e12*max(t)*0.005,2*yield,sprintf('Photolysis quantum yield: \n%.2e',yield))
```

4. Atomic dipole moment corrected Hirshfeld (ADCH) charge analysis

Table S2. The ADCH charges of the CS_2 units, R groups and Z groups of the S_0 states of different CTAs.

	CTA-*a*	CTA-*b*	CTA-*c*	CTA-*d*	CTA-*e*
CS_2	-0.11	-0.17	-0.15	-0.11	-0.12
R	0.05	0.05	0.03	0.07	0.02
Z	0.06	0.12	0.12	0.04	0.09

Table S3. The ADCH charges of the CS_2 units, R groups and Z groups of the T_1 states of different CTAs.

	CTA-*a*	CTA-*b*	CTA-*c*	CTA-*d*	CTA-*e*
CS_2	-0.10	-0.15	0.23	0.17	0.07
R	0.05	0.03	0.03	0.10	0.04
Z	0.06	0.12	-0.27	-0.27	-0.11

5. Experimental data

Table S4. The photoiniferter RAFT polymerization results for different CTAs.[14]

Monomer	CTAs	Conv. (%)	k_{app} (h^{-1})	*Đ*	Monomer	CTAs	Conv. (%)	k_{app} (h^{-1})	*Đ*
MA	CTA-*a*	72	0.51	1.93	NVP	CTA-*a*	11	0.03	-
	CTA-*b*	56	0.34	1.03		CTA-*b*	6	0.02	-
	CTA-*c*	88	3.36	1.12		CTA-*c*	46	0.19	1.25
DMA	CTA-*a*	70	0.39	1.82	MMA	CTA-*c*	97	-	1.96
	CTA-*b*	34	0.12	1.07		CTA-*d*	99	-	1.85
	CTA-*c*	96	1.53	1.14		CTA-*e*	32	-	8.41

Notes: Photoiniferter RAFT polymerization conditions: target DP=500, [Monomer] = 4 M, DMSO as solvent, blue LED light (465 nm, 17.0 mW/cm^2), 30 min Ar deoxygenation. Monomer conversion determined by ^{1}H NMR spectroscopy in DMSO-d_6. Molecular weight and dispersity determined by DMF GPC equipped with refractive index and light scattering detectors.

6. Further computational results on the chain transfer process

Table S5. Activation enthalpies and entropy contributions, as well as reaction enthalpies and entropy contributions of the chain transfer processes of different monomers with pyrazole-based CTAs.

CTAs	ΔH^{I}_{CT} kcal/mol	$-T\Delta S^{I}_{CT}$ kcal/mol	ΔH^{II}_{CT} kcal/mol	$-T\Delta S^{II}_{CT}$ kcal/mol	ΔH_{CT} kcal/mol	$-T\Delta S_{CT}$ kcal/mol
CTA-MA	15.2	0.5	-0.7	14.7	-15.9	14.2
CTA-DMA	15.2	0.1	-2.5	14.3	-17.7	14.2
CTA-MMA	12.5	0.0	0.9	13.6	-11.6	13.6
CTA-NVP	13.2	0.6	-4.8	14.1	-18.0	13.5

Notes: Quantities labeled "I" denote the activation enthalpies and entropy contributions of CTI fragmentation, while quantities labeled "II" denote those of CTI formation. ΔH_{CT} and $-T\Delta S_{CT}$ denote the reaction enthalpy and entropy contribution of the formation of the CTI, respectively.

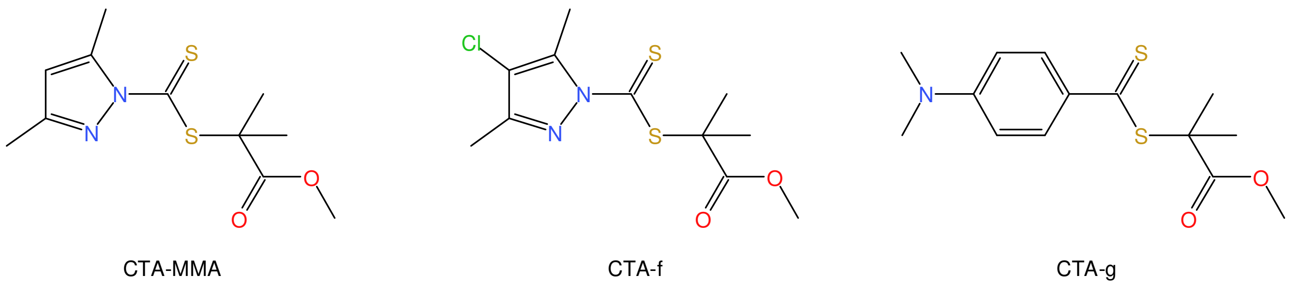

Figure S2. Structures of CTA-MMA, CTA-*f* and CTA-*g*.

Table S6. Chain transfer properties of CTA-*f* and CTA-*g* with MMA. The values of CTA-MMA (Table 3) are shown for comparison. For the meanings of different symbols, please refer to the footnote of Table 3.

CTAs	ΔG^{I}_{CT} kcal/mol	k^{I}_{CT} s^{-1}	ΔG^{II}_{CT} kcal/mol	k^{II}_{CT} s^{-1}	k^{II}_{CT}/k^{I}_{CT}
CTA-MMA	12.5	4.4×10^{3}	14.5	1.5×10^{2}	0.034
CTA-*f*	12.3	5.9×10^{3}	12.5	4.3×10^{3}	0.74
CTA-*g*	15.3	3.8×10^{1}	15.8	1.8×10^{1}	0.46

7. Optimized coordinates

All units are angstroms.

CTA-a S_0

C	-2.47020971095650	-1.49226311259107	2.71620113865414
S	-3.56116047131555	-0.48813583662434	3.42796618929626
S	-1.93183010318741	-3.04817899618659	3.38284766749668
C	-2.89040585041782	-3.13424115486282	4.93058890257807
C	-2.57019445460663	-4.39510801583182	5.60136613184145
N	-2.32314298534605	-5.38989069513175	6.13551117799082
H	-2.63417873280854	-2.29045849309802	5.59569746489169
H	-3.97382332287993	-3.09883019441201	4.71857080228762
C	-0.94445575352222	-2.01125634161798	0.87936006806625
C	-0.55468782903751	-1.31216661066505	-0.40468747267800
H	-1.40248785324667	-2.99785640489776	0.67375104434503
H	-0.06705981417647	-2.15111770802868	1.53971438532331
H	0.18434415660407	-1.93363348728722	-0.93916157092135
H	-1.42660479947547	-1.17103684325412	-1.06654944294776
H	-0.09480067891101	-0.32918357669201	-0.20351611607377
O	-1.91799666671626	-1.17623644881873	1.56556633984952

CTA-a T_1

C	-3.47106686393932	-3.23231787987688	2.22926718157155
S	-5.19821993694363	-3.17624003742223	2.12012280147242
S	-2.97161979872282	-4.23501187461265	3.58251468071579
C	-2.84558056950373	-3.05615597628100	5.00547202366099
C	-4.13542813608614	-2.74243003507462	5.61452703104521
N	-5.15882007880950	-2.50111429218728	6.09755443148145
H	-2.19739716624974	-3.54291188248363	5.75654220556817
H	-2.34656465158583	-2.12700187552302	4.67933310079155
O	-2.75894636887995	-2.18547511755844	1.78072731522389
C	-1.42041746075435	-2.45738723081538	1.30969238886493
C	-0.83054698793020	-1.16881366672523	0.77559878626993
H	-1.47115717764936	-3.23710424289391	0.52427367604400
H	-0.80810119484664	-2.85551825348504	2.14267229837993
H	0.19518244192252	-1.35720997309106	0.41326997319219
H	-1.42410659009412	-0.77300249677372	-0.06731974269143
H	-0.77729090869928	-0.39564907373702	1.56247429213411

CTA-a T_1 TS

C	-3.57099824950845	-2.90382099023805	2.17982729842643
S	-5.27191056701343	-2.71036402256624	2.16934168239544
S	-3.11726373506326	-4.10340830135884	3.25505208170187
C	-2.84481104535259	-3.15133404668399	5.25420592902164
C	-4.12177184694689	-3.00878081514636	5.87599967391496
N	-5.17976056398035	-2.90955930635181	6.34871105157321
H	-2.18750812476942	-3.89023452719706	5.73607545984824
H	-2.34763066857635	-2.20084509172919	5.01328420881785
O	-2.81233298444735	-2.08341445378757	1.48473719178692
C	-1.37596215865544	-2.29171047568278	1.43817732360292
C	-0.78157999163334	-1.25212524048458	0.51288011751399
H	-1.17995999295296	-3.31893788147537	1.07606969278418
H	-0.96842383173235	-2.20079649326913	2.46260569484284
H	0.31188711208721	-1.39596988803190	0.46778044940927
H	-1.18153405407045	-1.34739791524039	-0.51166941568456
H	-0.97633829738459	-0.22841255075673	0.87749056004478

CTA-b S_0

C	-3.29677238538279	-1.71889946928894	2.84642604629119
S	-4.85662131547241	-1.62103965881209	3.35813475139056
S	-2.00426064915056	-2.57861176726798	3.66596348086903
C	-2.87221677434296	-3.26260423571289	5.11716294630842
C	-1.91468541080910	-4.04086434874973	5.90275865778291
N	-1.16171903326406	-4.65754386346233	6.52626956871128
H	-3.28529681250353	-2.45070894423358	5.74276540398214
H	-3.70285533707884	-3.91823951791302	4.79900234182489
C	-1.06638941211514	-1.22102940015049	1.09328261739293
C	-0.64807099791144	-0.52844176500278	-0.20903482972835
H	-0.89153147985304	-2.31069186594727	1.02555452509133
H	-0.49178625967424	-0.81872481648735	1.94804312059809
C	0.84163391215415	-0.73950781234414	-0.51027891042297
H	-1.25562841881366	-0.91864527669535	-1.04953732700413
H	-0.86269453476584	0.55645711522840	-0.13946539687820
C	1.29314371576897	-0.06669657276282	-1.81238693925505
H	1.44466967604190	-0.35116162603307	0.33550196820079
H	1.05366243772495	-1.82673753839762	-0.56340424987833
H	0.68547688431906	-0.45609187806639	-2.65515201140406
H	1.07377352268058	1.01964775589048	-1.75877048108075
C	2.78134385579859	-0.27019271813264	-2.12263926626462
C	3.23517002760963	0.39480133867453	-3.42831140439926
H	3.00039771464314	-1.35717577387458	-2.16961013664927
H	3.38829864756254	0.12250249830697	-1.28056761026304
C	4.72362157247959	0.19339395711704	-3.73962390588619
H	2.62825612940496	0.00073932014717	-4.26989029993515
H	3.01417759171870	1.48150535484908	-3.38208774533447
C	5.17640490844046	0.85376051098217	-5.04810610953049
H	5.33064163873443	0.59049787743301	-2.89951264401021
H	4.94508854815811	-0.89339891495642	-3.78223508901681
H	4.57009460874715	0.45515045781378	-5.88804732471180
H	4.95320231585950	1.94024546634533	-5.00637232977767
C	6.66521038866206	0.65422411405605	-5.35909358237529
C	7.11714731766323	1.31126320285374	-6.66942071434603
H	6.88871329504139	-0.43232639628647	-5.39823355818910
H	7.27152097026102	1.05501721210323	-4.52016273654590
C	8.60665862259194	1.11441253707468	-6.98044825638631
H	6.51222593960075	0.90915699585166	-7.50892127657365
H	6.89227097691738	2.39772639266181	-6.63162298568935
C	9.05093258664976	1.76991080973506	-8.29034330997576
H	9.21147719177279	1.51808104478972	-6.14303759671136
H	8.83232108224387	0.02915214012296	-7.01682980320944
H	10.12787200102318	1.60928966508581	-8.48053470151508
H	8.49889061751998	1.36032401731412	-9.15745837050578
H	8.87828206003710	2.86280340863938	-8.27689562147554
S	-2.85097882669347	-0.91170318249820	1.36611402648604

CTA-b T_1

C	-3.71350560131999	-3.28275087167022	2.93566980028373
S	-4.68764941681962	-4.27677913700942	1.95636215227082
S	-3.44096461544243	-3.55422868565978	4.63710262770439
C	-4.27757525924752	-2.09624803006942	5.38206094660259
C	-5.72924427089509	-2.14025385361490	5.21608146667480
N	-6.87506256541251	-2.18838412125750	5.06323967384440
H	-4.02878428397570	-2.10374934665324	6.45892242874952

H	-3.87883263926626	-1.16149912945042	4.94991533634504
C	-1.19700776473029	-2.70590842528953	1.82663764417795
C	-0.33639623277278	-1.73564904201596	1.01518885542478
H	-1.23735638531906	-3.70411653698424	1.35370876883309
H	-0.80728587653921	-2.82395258083704	2.85434202575475
H	-0.76988005602874	-1.61047092167601	0.00287492768350
H	-0.35540192878345	-0.73496667393355	1.49147077987916
S	-2.91776148360518	-2.07120017303681	1.95022676035537
C	1.11520999531355	-2.21965173653291	0.89620160156167
C	2.00675439796771	-1.26515576295911	0.09231587576865
H	1.12857670408054	-3.22333619242865	0.42444523696727
H	1.54011589435545	-2.35440599526533	1.91178604394629
H	1.57740606895282	-1.12958012320975	-0.92183732221302
H	1.98748528926081	-0.26133495593046	0.56479855379714
C	3.45970444484702	-1.74082537271135	-0.03034275051443
C	4.35562046243894	-0.78694118271697	-0.83053518615861
H	3.47777967066976	-2.74453580446056	-0.50360562841588
H	3.88657265469865	-1.87948418538880	0.98469279143605
H	3.92844178775922	-0.64801371086633	-1.84547635678150
H	4.33673116547870	0.21672400326699	-0.35703191097618
C	5.80910006375251	-1.26150102666353	-0.95335198437802
C	6.70614910555723	-0.30671772109793	-1.75129979868025
H	5.82792124940612	-2.26460419296084	-1.42811323166407
H	6.23545814143230	-1.40206009068753	0.06174846555201
H	6.27941592188075	-0.16562450111893	-2.76617887569284
H	6.68749202905548	0.69615545250869	-1.27600733580876
C	8.15957669692906	-0.78143093180561	-1.87469765696266
C	9.05723487258345	0.17397675807379	-2.67104198723352
H	8.17804939344057	-1.78385394782153	-2.35099280785223
H	8.58573083071826	-0.92371981751493	-0.85971328138550
H	8.63090481053258	0.31747290378351	-3.68594445905240
H	9.03992905268844	1.17638265682348	-2.19430949944108
C	10.51088735070585	-0.30059151284933	-2.79639457408314
C	11.40183911408160	0.65743990344079	-3.59086293994408
H	10.52877183949933	-1.30145396756840	-3.27384428594614
H	10.93720175584002	-0.44469211869623	-1.78273203371015
H	12.43951206398538	0.28330747550418	-3.66163694034446
H	11.03042100086684	0.79541659108721	-4.62401427776891
H	11.44395876844365	1.65793318208062	-3.12007567201866

CTA-b T_1 TS

C	-4.07511211449806	-3.02113484990456	2.39218361497643
S	-5.77852354826997	-3.03408455636508	2.47105694896733
S	-3.47725134553764	-3.99296459225061	3.61453106066334
C	-3.23678180759038	-2.56328346319040	5.47241889539385
C	-4.49938875586554	-2.49628421256121	6.11837452483531
N	-5.54739900405961	-2.46308760536007	6.62436177752124
H	-2.45473692078937	-3.11849813212589	6.00867138251813
H	-2.90999822396436	-1.64480771997576	4.96620137823792
C	-1.46591871748334	-2.45751519873725	1.56279435931206
C	-0.59039270286630	-1.70535156051110	0.55530056044548
H	-1.34471569923496	-3.55099483723404	1.45672645848507
H	-1.20852339999651	-2.17469174144234	2.59948498261737
H	-0.88105098009147	-1.98887397571642	-0.47558138941317
H	-0.76745933739435	-0.61524468086796	0.64606144749648
S	-3.22014483040183	-2.03842557177518	1.25907620973488

C	0.89961138607594	-2.00201996677538	0.77081726390232
C	1.80858222208628	-1.27678899729393	-0.22908975966251
H	1.06949220143614	-3.09558408783242	0.69890177207624
H	1.18352723252541	-1.71572780039237	1.80394708423184
H	1.52457662844301	-1.56963138743332	-1.26095512598976
H	1.62635886576158	-0.18419262629214	-0.16425481913050
C	3.30118012537592	-1.55634278139835	-0.01377766269306
C	4.21343342220434	-0.84092400347158	-1.01821330082503
H	3.48108774672031	-2.65007171300213	-0.06963846864634
H	3.58452550204470	-1.25733041776879	1.01677743136794
H	3.93329868723562	-1.14504675870599	-2.04824006042657
H	4.02833260675540	0.25226254297665	-0.96722856274334
C	5.70709367550019	-1.11226662270567	-0.79844300334800
C	6.61943792167568	-0.40272598538836	-1.80705767798226
H	5.89145983654944	-2.20585471481005	-0.84401949981370
H	5.98776319041118	-0.80333540125791	0.23004244040344
H	6.34108106131683	-0.71497610048382	-2.83518607294458
H	6.43208455957610	0.69049064700580	-1.76451633864694
C	8.11350302480595	-0.66931375350085	-1.58391792720383
C	9.02604529469525	0.03689085550434	-2.59457384225647
H	8.30034561220609	-1.76275278664902	-1.62352333136406
H	8.39199227732973	-0.35442712819523	-0.55660396366783
H	8.74910843342090	-0.27921259632955	-3.62209115062721
H	8.83840708102787	1.13040053749140	-2.55665697248823
C	10.52078565643930	-0.22711125775136	-2.37064907343732
C	11.42625013815977	0.47907704804704	-3.38271276075684
H	10.70876421752699	-1.31950418991243	-2.40793095612263
H	10.79822706966085	0.08964043714293	-1.34462427752657
H	12.49375100744304	0.26591473665938	-3.19090387010714
H	11.20705714511514	0.15690231684746	-4.41836835776505
H	11.29799755851871	1.57753465369355	-3.34583536759774

CTA-c S_0

C	-1.97713239779191	-0.61578020956531	-0.80876422219266
C	-2.39542804530454	-0.17260460026882	0.41855930036180
C	-2.82967319077633	1.20767828224743	0.79218914599259
C	-1.67100057250658	-2.00787301853548	-0.65808829433360
H	-2.76137542883619	1.84363324281581	-0.10536035110826
N	-1.88628725795742	-2.39328543780682	0.57737493120609
N	-2.32887609059820	-1.29414979440653	1.25672121130088
C	-2.62584094491623	-1.45730677340300	2.60376629355260
S	-3.17097254750192	-0.27927402913568	3.60492967619111
S	-2.32819614947574	-3.13715230435866	3.08846447564209
C	-2.78169169839942	-3.05192876875089	4.85675757524487
C	-2.58868240517799	-4.38325161662532	5.43327260703155
N	-2.43937715668936	-5.43445311981612	5.89065543140617
C	-1.17396601142367	-2.95418635614567	-1.70483314089871
H	-1.02931750210785	-3.96181493121530	-1.28266939181817
H	-1.89041701424051	-3.02092026164056	-2.54269565936622
H	-0.21357867648117	-2.60442693217314	-2.12322244592704
H	-1.89747904516177	-0.01447429620567	-1.71765883317461
H	-2.19166970315925	1.64421090172977	1.57916172925059
H	-3.87010213592586	1.23041828555140	1.15794014632132
H	-2.14478261645349	-2.32922579274296	5.39789864769234
H	-3.83753275911458	-2.75315770954847	4.98551188762524

CTA-c T_1

C	-1.72373799843378	-0.33155780916517	0.14547061869055
C	-2.82152658908346	-0.20752572446578	0.97207853389672
C	-3.92830475548594	0.79831731938481	0.99979825681238
C	-1.03886367703179	-1.50335273672622	0.58547233089677
H	-4.91859389795526	0.30884310993352	1.02359066101928
N	-1.66573963488832	-2.05466628446680	1.61181881946494
N	-2.74728653527059	-1.26670403705496	1.84847723918808
C	-3.64499607843874	-1.60226255100697	2.86922094674250
S	-3.92584417661693	-0.64618745014951	4.26021527532912
S	-4.46170331203072	-3.13728875775558	2.96138447968875
C	-3.16588290129393	-4.28536821444663	3.60486800624880
C	-2.88661844002750	-4.09586116888517	5.02568829655541
N	-2.66505512058280	-3.93441150978044	6.14963031812734
C	0.21864788297840	-2.09883021289103	0.02617310747207
H	0.49926063791207	-3.00739585395553	0.58435073097958
H	0.09224133420193	-2.36902762247739	-1.03751286511893
H	1.05772397170855	-1.38262479248550	0.08326838343675
H	-1.45074659011419	0.32994577976999	-0.68051578908835
H	-3.87794304215275	1.42959187840090	0.09846827176330
H	-3.85969899625249	1.46436561465634	1.87935780316309
H	-3.55107312377376	-5.30789657667037	3.43829515406642
H	-2.24406407005539	-4.15041426038726	3.01289370882508

CTA-c T_1 TS

C	-1.56762578997896	-0.27284268692146	0.53633332291713
C	-2.77750964337823	-0.28315878573187	1.18185760471247
C	-3.66040386369291	0.86249884720519	1.56019934276812
C	-1.15892639349375	-1.64291601793364	0.43480448162424
H	-4.66980777413519	0.77600991993675	1.11965625718022
N	-2.05513532571516	-2.43986106232943	0.97563940460126
N	-3.04816830794469	-1.62665286508229	1.43613444274330
C	-4.11564152628812	-2.21107067591219	2.08684124907006
S	-5.40005970397262	-1.45867817428728	2.89631009244317
S	-4.27343034442349	-3.85663832262838	2.33242717363283
C	-2.98571255791510	-4.31078451504199	4.21537626456589
C	-2.35110594282537	-3.08003011492253	4.53179442062912
N	-1.88184173027092	-2.03144135670923	4.71748409505088
C	0.09460396567967	-2.17847355000804	-0.18342307254694
H	0.12630186589211	-3.27756050615355	-0.10745971786446
H	0.15839060646681	-1.89659313867610	-1.24949207480793
H	0.98745979349394	-1.76422121031648	0.31777051726823
H	-1.03261540925521	0.60949668274485	0.17655169870239
H	-3.20894699941120	1.79412432335207	1.18280636920604
H	-3.76333091430769	0.96082904613175	2.65596359908735
H	-3.75368062352764	-4.64596961115512	4.92566869917491
H	-2.32765838099625	-5.09443122556103	3.81610482984170

CTA-d S_0

C	-1.55624578854343	-0.59973247632349	0.08509470419599
C	-2.41671648099352	-0.47979622635665	1.14556161862664
C	-2.87218958282704	0.78331454175491	1.80314253104314
C	-1.43580372107879	-2.00106942728405	-0.18449141993159
H	-3.96763750301045	0.90369412950480	1.75738751805279
N	-2.17229015522460	-2.69432033337880	0.65126529731441
N	-2.78185501252107	-1.79205001909722	1.47575127995555
C	-3.62638865745984	-2.29216690012428	2.46619710152968
S	-4.40048340669524	-1.36063617444796	3.57645242068371

S	-3.72320734845024	-4.05137408623022	2.36837742721190
C	-4.92775036207958	-4.52467000522917	3.66048908719798
C	-6.38651750088753	-4.26426988322730	3.28481582540663
H	-6.61531355462195	-3.18744103942982	3.22556976188044
H	-6.61634991525483	-4.72777293853116	2.31063914314396
H	-7.05288016174674	-4.71455946762986	4.03985585941358
C	-0.61259181557332	-2.66236931125437	-1.24501809797393
H	-0.72543686322452	-3.75772219788787	-1.19924537275125
H	-0.91726908116981	-2.31749536969294	-2.24907818153085
H	0.45627402726566	-2.41182653506451	-1.12415001254842
H	-1.06667143020973	0.22225173288146	-0.44286318836872
H	-2.40520896183239	1.63115046291831	1.27563846297846
H	-2.57892659681039	0.82803275214906	2.86539201682738
C	-4.57318161581711	-4.09789634565293	5.09124831868966
O	-5.40091634980014	-3.91263348179071	5.94923374204787
O	-3.25725452373684	-4.07330316281444	5.30329934240422
C	-2.81201721331132	-3.70573057110117	6.61206608376222
H	-3.14780912607403	-2.68620908574690	6.87278256354377
H	-3.17670642692890	-4.41559503275089	7.37529367830553
H	-1.71210604394672	-3.73181624018605	6.58257183414176
H	-4.76482070943547	-5.62413104497570	3.65939010674737
CTA-d T_1			
C	-1.24392120397802	-0.74710733403630	0.23483540413458
C	-2.23035546948542	-0.42673615743800	1.14708273129955
C	-2.52444754800731	0.86609247893481	1.84017520979725
C	-1.41505643975326	-2.13342109979640	-0.04594471511860
H	-3.47679652279072	1.31626798994959	1.50485076863563
N	-2.43692874283171	-2.62726269217364	0.63639544632937
N	-2.93440345926655	-1.59121093914492	1.35750705866758
C	-4.01661693369190	-1.80669860204885	2.22148824870452
S	-5.41877815386026	-0.83112613743039	2.35094590114415
S	-4.25024210708143	-3.27201395695560	3.12778996748695
C	-3.25014814068452	-3.04712497053069	4.68113016139192
C	-3.64197580003849	-1.81638644379017	5.49003806876309
H	-3.47950913501534	-0.87946252467049	4.93085809125902
H	-4.70488598964045	-1.87229561877415	5.78125935657113
H	-3.03950183439850	-1.76883589009578	6.41506628189059
C	-0.60285695014713	-2.99677013650810	-0.96517706040840
H	-0.98438450013870	-4.03132911105159	-0.96081671063176
H	-0.63587585917842	-2.61865608596054	-2.00266264884556
H	0.45851131232160	-3.01697287096426	-0.65888387650727
H	-0.48635785444664	-0.07073177409047	-0.16907372228742
H	-1.72149328361902	1.58766685402774	1.61949745753065
H	-2.57648636597074	0.73744771930328	2.93601542103835
C	-1.77183593038205	-3.15994063183016	4.30877881726442
O	-1.18027475174661	-4.20849777238477	4.22191030603716
O	-1.22129367778176	-1.96780756845139	4.07302320278220
C	0.14823511396682	-1.94457378797046	3.65196097112184
H	0.80939213619247	-2.34804612019484	4.43908602191031
H	0.28969758793991	-2.52777744875245	2.72531515839992
H	0.39899943362602	-0.88878088013989	3.46993125277285
H	-3.49996399483004	-3.97181074569812	5.23370582161926
CTA-d T_1 TS			
C	-1.38456938692098	-0.47299603544337	0.09714750541039
C	-2.44440395531568	-0.10718530314925	0.89016266591678

C	-2.97145656609158	1.26184850363710	1.18240259245546
C	-1.30822834759607	-1.90006806483389	0.17226638869443
H	-4.01121204621187	1.39281313804525	0.83231151340178
N	-2.25340215226181	-2.37755201756294	0.95487227471922
N	-2.95422910588636	-1.29817658283702	1.39897108959433
C	-4.01428804307871	-1.51875973643649	2.26431884700036
S	-5.10677267681094	-0.36731343814212	2.88016030717582
S	-4.39968972208741	-2.99550013021611	2.93378471698873
C	-3.15913461901413	-3.25367908147044	4.92846291449816
C	-3.41089383732239	-2.02002139889488	5.74422763905165
H	-3.09013470784381	-1.09833403979351	5.22871712509829
H	-4.47829572945107	-1.92752481492884	6.00735245436465
H	-2.84231901017875	-2.07270172449060	6.69557296385113
C	-0.32357727579499	-2.80263852429113	-0.50487735147377
H	-0.52017194536696	-3.85594303855603	-0.24605978891226
H	-0.38013469526785	-2.69524301063237	-1.60288999698279
H	0.71029650731914	-2.55276096890752	-0.20687143731337
H	-0.73723279541441	0.20067559651124	-0.46968197561471
H	-2.34669053849861	1.99872444202096	0.65221016388217
H	-2.93343861112187	1.50214510074802	2.25975588432596
C	-1.81558601867141	-3.53999547614075	4.37021358666200
O	-1.40282770816137	-4.65343144359145	4.11716344837302
O	-1.10378379770183	-2.41974823261507	4.15506554484878
C	0.19321352003799	-2.56813382864889	3.57664000186456
H	0.90390875616584	-3.00479996639034	4.30213611702458
H	0.16608290812630	-3.20579561141704	2.67635865203535
H	0.53117696418782	-1.55641934424689	3.30272363042273
H	-3.67989736376617	-4.17074696732560	5.24124652263651
CTA-e S_0			
C	-2.01421298262972	0.10603197405821	0.56413789070379
C	-3.26661088404794	-0.10439254083157	1.08366039310202
C	-4.40585188927995	0.85801442741384	1.20251393572699
C	-1.29218413591613	-1.11596934234911	0.75116594675321
H	-5.21034903887611	0.63865791962612	0.48061882789694
N	-2.03905476816639	-2.01039438964405	1.36218820900020
N	-3.24959383926896	-1.41159764958283	1.56754953753580
C	-4.28137827244933	-2.14959627689397	2.14259099972195
S	-5.84971070550655	-2.03406704961726	1.68795417908602
S	-3.88385777871562	-3.39845239646911	3.32175511566671
C	-2.51338228113477	-2.80894730717055	4.48456474065917
C	-3.00752058769437	-3.35581388942307	5.77044409072175
N	-3.37272793499010	-3.77854633063375	6.78389846189436
C	-2.44406935754419	-1.27559003406084	4.61831121590564
H	-1.96706648945390	-0.82510735156316	3.73401568777725
H	-3.44072292293217	-0.82603171866270	4.76661044540790
H	-1.81897390947486	-1.02342735066554	5.49277893532366
C	-1.13064831978342	-3.42846540280242	4.16506646896592
H	-0.44568991802513	-3.08997453764095	4.96680010871889
H	-0.78053621085454	-2.96425811258829	3.22859625730588
C	-1.08708580391083	-4.95023998186897	4.04833217098138
H	-0.04848700375222	-5.28113213428300	3.87358401913665
H	-1.44032336263234	-5.45495034448155	4.96693915441766
H	-1.69372318611047	-5.31648976185109	3.20060030940492
C	0.11951639610443	-1.41545818554620	0.35204450310041
H	0.35742071890052	-2.47847157764835	0.52044365525350

H	0.28570154242469	-1.18159028194987	-0.71399501397027
H	0.82851376281314	-0.80147561953330	0.93617976567382
H	-1.64861275193765	1.03074151899109	0.11104009548539
H	-4.02909729086050	1.87364208521224	1.00008781478257
H	-4.84744477529443	0.84959086046022	2.21426636085963

CTA-e S_1

C	-1.40206782872168	-0.52682599117641	0.10646969578661
C	-2.40987962733360	-0.36555586271764	1.03709292495678
C	-2.94901265964696	0.87658812563049	1.67269103479707
C	-1.29883214425155	-1.93088064984504	-0.11224309391859
H	-3.97077769461800	1.11250420544320	1.32310017117271
N	-2.18407570746703	-2.58543446507983	0.62420368830440
N	-2.85886800191143	-1.63640067750088	1.32099490839952
C	-3.86674329200955	-2.02967714851598	2.21178865755786
S	-5.40657472734893	-1.35704442033388	2.40852100092999
S	-3.76354343589155	-3.45370189748612	3.17397789060579
C	-3.08611229593362	-2.89330430413269	4.88069975398041
C	-2.83255430456686	-4.18817841775632	5.54131509023164
N	-2.63993114147866	-5.20846966863519	6.05238695876881
C	-4.14908865765729	-2.10808655413165	5.66341408891334
H	-4.34256579739286	-1.14170984670765	5.16805085613440
H	-5.09722650884253	-2.66505130208538	5.74614289845034
H	-3.77462642946329	-1.89924734493410	6.68155499377362
C	-1.78082962066275	-2.08495483873738	4.71113271400924
H	-1.47744913027375	-1.76386069923571	5.72628532805120
H	-2.04778316774612	-1.15654355181073	4.17076111768720
C	-0.61947480860159	-2.79651602714166	4.02030840054951
H	0.24813565596971	-2.11700216195193	3.95826254876435
H	-0.29410887647616	-3.69452597249695	4.57675026742827
H	-0.86922010287823	-3.10663801929803	2.98954149292213
C	-0.35243423564591	-2.65700703514427	-1.02084163449241
H	-0.53825702700406	-3.74324117869888	-0.98455839270585
H	-0.46383012405379	-2.31917164794277	-2.06657044673619
H	0.69750282946947	-2.47455963866490	-0.72906608553742
H	-0.80692333570978	0.26708028802455	-0.35162379484872
H	-2.30188093559269	1.72889784787010	1.41026660150376
H	-2.97873084725886	0.79475807319378	2.77393464756008

CTA-e S_1 TS

C	-2.84007825964403	-0.20069901997160	0.16764750766228
C	-2.95557452296977	-0.58808800920013	1.54056228017009
C	-1.78409861786308	-0.21896932614231	2.13995936624281
N	-1.02186918689283	0.36595951956123	1.13164914434851
H	-3.80205326886511	-1.07499574541584	2.02176183495540
N	-1.68157261198297	0.37149023021826	-0.06049283826932
C	0.23265344130362	0.91554215742465	1.18088413486211
C	-1.36124268512085	-0.36098502324854	3.55993749943148
H	-1.13065265921783	0.61323147257056	4.01679591557556
H	-2.18477603047627	-0.81653698347491	4.12518444351828
H	-0.47750285083661	-1.00843802739538	3.66225740722347
C	-3.84688862385916	-0.38840404914977	-0.91467402812915
H	-4.05312941751773	-1.45818155548365	-1.07038853896186
H	-4.79939934062971	0.09223164336053	-0.64529145060050
H	-3.48462713721349	0.04335798702448	-1.85700867393802
S	1.35026756316659	0.88483943495640	2.44411300722983
S	0.95693118237169	1.71116631843249	-0.09408082535619

C	1.97980153500114	-0.09225254044141	-1.50296850491938
C	1.13925654718828	0.10341697406237	-2.73231033894009
C	1.37966785260438	1.38650572442959	-3.51312436808931
H	0.72496941752629	1.42420323147973	-4.39591989192433
H	2.41988724711179	1.46361528580295	-3.86558170954850
H	1.31605911409181	-0.77183379447660	-3.39097136950459
H	0.08097501059971	0.02487737936821	-2.42869752599215
C	3.28944969454772	0.43648265757509	-1.48631942550173
N	4.35544725403933	0.90979930236716	-1.44590660188419
H	1.16310230090746	2.27908469067188	-2.90426535370515
C	1.70403195894004	-1.28163740132546	-0.64524785557163
H	0.62320148712647	-1.38498601689024	-0.45999182707799
H	2.02796184604776	-2.20466543934528	-1.16262981234592
H	2.23376272051547	-1.23886433734458	0.31666964904022
CTA-e T_1			
C	-1.42689907722244	-0.46707628387684	0.23297112075243
C	-2.46328334256132	-0.39210543110826	1.14149274748113
C	-3.06320800507391	0.78567491666599	1.84245619507936
C	-1.28334683069730	-1.85191285593647	-0.07455039651767
H	-4.07370198108828	1.02426173087410	1.46322575005862
N	-2.17073101346041	-2.57505875203692	0.59024142650611
N	-2.88660857023909	-1.68945285312876	1.32985665739117
C	-3.91514451558018	-2.14859145984702	2.16615327471684
S	-5.55007911120203	-1.65073903614562	2.07561823619223
S	-3.77026524736140	-3.53116323398956	3.20528095549540
C	-3.07025599270327	-2.83341145108735	4.82242433163910
C	-2.96955821230646	-4.04205139696422	5.66523083833887
N	-2.89363287299215	-4.98963590279856	6.32502332579113
C	-4.05459902568546	-1.84084932132111	5.46092976260975
H	-4.16354619604452	-0.95241316075838	4.81541802578963
H	-5.04862978707651	-2.29170011623430	5.61924211431898
H	-3.66187878973139	-1.50382677244171	6.43671035288977
C	-1.67322785286500	-2.19970119133147	4.61644594860593
H	-1.34504592103489	-1.83573657996634	5.60927969829060
H	-1.81717935125450	-1.29465472443515	3.99703112425769
C	-0.59831344042959	-3.09888325856469	4.00952292549798
H	0.35520361435930	-2.54647888577590	3.94413964512230
H	-0.41429698492790	-3.99926723830937	4.62437666203267
H	-0.85966029174379	-3.42934696974227	2.98853908266722
C	-0.29548460701453	-2.49260417998427	-1.00346127116882
H	-0.45642395047640	-3.58257535236644	-1.04687847436331
H	-0.38728126711876	-2.08587191548054	-2.02633296081664
H	0.74173951101403	-2.30796065136673	-0.67054080295589
H	-0.83888405205191	0.36853419372009	-0.15482817647070
H	-2.42868491587711	1.67138416036016	1.67939930674784
H	-3.14299036039518	0.61604770457465	2.93119635062311
CTA-e T_1 TS			
C	-1.34895888291548	-0.56274532663567	-0.03716250656316
C	-2.35125312158574	-0.23861349450908	0.84570077277439
C	-2.82897628643582	1.11113691962278	1.27856151430509
C	-1.29103920100631	-1.99114163718679	-0.06136092747737
H	-3.86844711544135	1.30982805169745	0.96025955341971
N	-2.19202141426191	-2.50916727477810	0.75000767571185
N	-2.84559070968204	-1.45243116750898	1.30663492337996
C	-3.86375434308311	-1.71040025666085	2.21622484029708

S	-5.07548427107914	-0.63352515043396	2.74028672991862
S	-4.09919621773864	-3.16662124589964	3.00220254774330
C	-3.05795900398339	-3.02577588701368	5.07038809174184
C	-2.68875158918194	-4.41421297009384	5.19724180038996
N	-2.41973268747168	-5.54261633453476	5.26901280969403
C	-4.17521655247412	-2.58833084064798	5.99721212840893
H	-4.51350810144457	-1.57026993794655	5.73939756981948
H	-5.04185130766325	-3.26975438070310	5.96040579538817
H	-3.80667394566541	-2.56425584177160	7.04183743925442
C	-1.91257698066876	-2.04487875804901	4.88268582571552
H	-1.58228635803730	-1.73843493424312	5.89744159656543
H	-2.33006449465902	-1.12251744826150	4.43089875972537
C	-0.71351160580768	-2.54567325764897	4.07911653785848
H	0.01717032061128	-1.73014569265690	3.93853365324451
H	-0.18992409628517	-3.37094999700000	4.59497466092761
H	-1.00759237666868	-2.90667935071296	3.07708616545189
C	-0.36973444735584	-2.85874411210143	-0.86234198874753
H	-0.58214835643634	-3.92460172707896	-0.67809774102112
H	-0.47958359071716	-2.66000644362627	-1.94322764400238
H	0.68480417948083	-2.66093738550731	-0.59942429497390
H	-0.72618203053238	0.13988604141403	-0.59628583541946
H	-2.18861518375892	1.87750081828480	0.81281740012014
H	-2.76910722805094	1.24131902219199	2.37371814634909

CTA-e T_2

C	-1.36480503699499	-0.67624878950071	-0.45884051730363
C	-1.90248368316910	-0.34884190154255	0.76524879698841
C	-1.85746739615553	0.93175564161184	1.53554553402694
C	-1.65474319269291	-2.06233141771656	-0.65491673979250
H	-2.84380858989998	1.42716055213565	1.57264381027841
N	-2.32711748119904	-2.54914686464338	0.37583023460019
N	-2.48657595714684	-1.51537519223283	1.23737720062439
C	-3.11938326547184	-1.71483977834433	2.47375092196104
S	-4.56199643043866	-0.71680106071679	2.76387703976503
S	-2.96238813544643	-3.33315108227509	3.09620406891187
C	-3.05913066392736	-3.10135995986455	4.96509764343497
C	-2.35240146803806	-4.30592592335577	5.45683698832969
N	-1.80775091516247	-5.24719822709949	5.85226548653072
C	-4.51579948384652	-3.14738022747647	5.46414187034998
H	-5.07527296797617	-2.27415187382077	5.09203264711527
H	-5.02938700867074	-4.06217437636604	5.12391977756087
H	-4.53366043984887	-3.12510934004397	6.56824984295023
C	-2.31818068143466	-1.82919653209683	5.45152438567743
H	-2.44044121646519	-1.80070636843004	6.55135144808374
H	-2.87468541207943	-0.95336766439009	5.06826612434926
C	-0.83809704842324	-1.71754585707488	5.09245005470167
H	-0.42804955829091	-0.77786971972359	5.50156999774906
H	-0.24563581103981	-2.54718096905431	5.51990083833503
H	-0.66658281939151	-1.70660489923617	4.00141643116463
C	-1.29072839486195	-2.92050557634539	-1.82656219488175
H	-1.66302970711120	-3.94836848379110	-1.68480453492131
H	-1.71970239165551	-2.51747833384472	-2.76139494590659
H	-0.19496579811336	-2.96042655262193	-1.96225666554630
H	-0.81079652416817	-0.01132541270483	-1.12601799596983
H	-1.14679410321882	1.62212489125622	1.05364228553426
H	-1.52590041766068	0.76580629930953	2.57639616529873

CTA-MA CTI

C	-1.65230394630010	-0.62108313900502	0.59107843219712
C	-2.61175840269151	-0.47783824583906	1.56811196440646
C	-1.76898951710909	-1.96316259200524	0.11887957756858
H	-0.93896667165366	0.14042334244628	0.26620455762714
C	-2.91050558655080	0.70548587193073	2.43390397935905
N	-3.24853726281084	-1.70422364878233	1.64508259103913
N	-2.72492319865221	-2.60444901628566	0.76586512273020
C	-0.96481120030047	-2.63640368388649	-0.95130430099068
H	-3.02151819423754	0.41853379663430	3.49381423199913
H	-2.07736063661409	1.42285131971642	2.35766858840725
H	-3.83533871183108	1.22142440177291	2.12384333263588
C	-4.34765985753253	-2.07742392371123	2.44023343552628
H	-1.29178710106279	-3.68101396331521	-1.08277530828736
H	-1.07226583087246	-2.11163476889887	-1.91728615815061
H	0.11020050143375	-2.63548138043367	-0.69715178776810
S	-5.67430820687127	-1.11589310796373	2.64071773027735
S	-4.38271686805429	-3.70389065542143	3.10317114352497
H	-2.00054064862071	-3.83813877766448	2.67394817845077
C	-2.63461359696829	-4.11511318081295	3.53116354141886
C	-2.22000309366128	-3.24468245837818	4.71575640759975
C	-2.55549596155453	-5.62247989597925	3.78037212021088
O	-1.59950790606678	-2.21347393659328	4.60266040684515
O	-2.64359638969939	-3.73544669200761	5.88024831358666
H	-1.53039853943308	-5.89412132384545	4.08846752900831
H	-3.24876447096297	-5.95643616387276	4.57135785943886
H	-2.78648872811710	-6.17533920605798	2.85358152886132
C	-2.35775341783483	-2.97631996610213	7.06159213362408
H	-1.27001018458065	-2.84931452494670	7.20061546586527
H	-2.83933466312086	-1.98344090214422	7.01995663546458
H	-2.77174974961085	-3.55048316702467	7.90412911846829
H	-7.75044202963394	-0.65011732913044	0.78825093131355
C	-7.06236639820079	-1.47085102489953	0.54314829459128
C	-6.07929653086481	-1.26499472203383	-0.56343410435237
C	-7.58422305041794	-2.80182019868909	0.90880943306623
H	-5.66208365248895	-0.24387822426702	-0.54038411783813
H	-6.58397742303305	-1.38872708907327	-1.54463392423192
H	-5.25103766053759	-1.99334361377887	-0.53329574191695
O	-8.61278691783985	-2.98845830525144	1.52844701929817
O	-6.78160659957999	-3.79756936485644	0.49184572997139
C	-7.14603693438610	-5.13023100727106	0.84909925431907
H	-7.27603616022415	-5.23720191965137	1.94074128198891
H	-6.32159310116103	-5.77852330914741	0.51399151831328
H	-8.08056449968948	-5.43845930347324	0.34602005453290

CTA-DMA CTI

C	-1.09512488258070	-1.29759473144753	0.18172348685014
C	-2.11067626035369	-0.54514793135289	0.73063664161053
C	-1.39035828520725	-2.65412493214992	0.50877045195449
H	-0.23507181921684	-0.91809987934056	-0.37556590290039
C	-2.29464586518937	0.94018163741301	0.73271199511067
N	-2.94985549160111	-1.45149004035281	1.35279042992062
N	-2.50206372414900	-2.72964220237347	1.21781999937592
C	-0.60849756038003	-3.88081180438953	0.14826307820052
H	-2.56556099397018	1.32038046478416	1.73343099812884
H	-1.34941689866068	1.41822494913136	0.42848155620351

H	-3.08140578662730	1.25847024488928	0.02736964098647
C	-4.16530888384394	-1.20342713296782	2.02510184315257
H	-1.09526656073498	-4.78555892536047	0.54848853193629
H	-0.52084926461167	-3.98759320978996	-0.94759804933650
H	0.41754321392119	-3.82840251994551	0.55415561353878
S	-5.34075772769562	-0.21678557980894	1.41211121439212
S	-4.51277056663282	-2.09816468646557	3.49217455781082
H	-6.44420935653819	-1.82000539584013	-0.70064641970482
C	-7.10317022169429	-1.48624238429711	0.10932170688055
C	-7.52264474977785	-2.45723101319659	1.15870129927231
C	-8.00880162851204	-0.33862028548740	-0.19922088340385
O	-8.47967378253293	-2.19897764814903	1.89068569472926
N	-6.79986805270122	-3.61832787136567	1.27950040155618
H	-7.48689442061123	0.44326687677052	-0.77805970163424
H	-8.85771643490864	-0.68693957794048	-0.82427374545631
H	-8.44181201901325	0.10506253866729	0.71293393828537
C	-7.18814240165354	-4.59893115032210	2.28011407752993
C	-5.76167685213202	-4.04342056140230	0.34934910764234
H	-6.32164949750329	-4.86810326757103	2.91415063587305
H	-7.56970485827052	-5.52614122249622	1.80786931045156
H	-7.97845453500192	-4.18226929865475	2.92029565412812
H	-6.18215685070479	-4.52691119699224	-0.55553972122836
H	-5.11150988778012	-3.21300257288838	0.03306787820416
H	-5.11247253665328	-4.77979142399682	0.85255318410218
H	-2.21392280285087	-2.87204230392386	3.56412328473591
C	-2.88541600645441	-2.42094718995909	4.31208445513538
C	-3.20386402332738	-3.44372117781306	5.43017930438019
C	-2.26794624975112	-1.14130640941479	4.86699980826435
N	-3.34762293494736	-4.75140417999265	5.06714566428258
O	-3.33859099591956	-3.05460939539514	6.58328588358459
H	-2.07352739259612	-0.41912659722596	4.05452383259313
H	-2.92039664300489	-0.66281128438071	5.61559545023278
H	-1.29941692370585	-1.36873955986440	5.34798920583415
C	-3.06624606728811	-5.29611497706007	3.74342122539344
C	-3.61950807993773	-5.74765084242608	6.09502426799792
H	-3.17755424100614	-4.54754477341699	2.94527172948424
H	-2.04201537657540	-5.71619360668038	3.68835778960541
H	-3.77765726630017	-6.11322276017479	3.52985579432220
H	-2.74194505772265	-6.40498635324443	6.25297442711139
H	-3.86091423437786	-5.25072663624448	7.04499867113655
H	-4.47363426071123	-6.38181521809350	5.79408070174292

CTA-MMA CTI

C	-1.55027131188320	-0.10419603190065	0.12878037911870
C	-2.63850088000580	-0.00233559779882	0.96829741254376
C	-3.35255945115319	1.22399378960965	1.44449697848281
C	-1.24761795407107	-1.49565773034754	0.04623786773626
H	-3.53720447407017	1.19716189800645	2.53274374105280
N	-2.08126941082199	-2.19857724441417	0.79229898686517
N	-2.93369344045758	-1.29813388578185	1.35129533079222
C	-3.98211251187646	-1.75215065675546	2.17656098224934
S	-5.53482140553612	-1.20638375569223	2.00103121804723
S	-3.71055930194734	-3.10337674965367	3.26506673240555
C	-2.03156646328082	-2.93228239668915	4.11209772893031
C	-1.50271929256804	-1.49684341725057	4.13168831672944
H	-1.12106712297105	-1.19592767073835	3.14333875993376

H	-2.26359637835301	-0.76926250138202	4.46199371641662
H	-0.65592984458750	-1.43919821997274	4.83790356829774
C	-0.15533824590939	-2.15753054064772	-0.73825570474593
H	-0.21207282107842	-3.25386483844339	-0.63569008704419
H	-0.22536376378181	-1.90284589425010	-1.81061120346626
H	0.83883912301426	-1.82610141230024	-0.38824498356944
C	-2.39512006705152	-3.36417218588213	5.55189174582236
O	-2.30179575276466	-2.65858567173246	6.52550080307301
O	-2.83247361994081	-4.62490298277512	5.58480012560001
C	-3.24764188764546	-5.15347510649752	6.85080611750270
H	-2.41058339159362	-5.16249190770912	7.57042788274912
H	-4.08487284622180	-4.56780185833269	7.26930197900015
H	-3.57946063556648	-6.18495828611719	6.65927924417026
H	-4.53408556112039	-3.41276656810120	-0.20905591519375
C	-5.57611992393995	-3.70780130542640	0.00829514693719
H	-5.89922232472086	-4.37531652838319	-0.81802399146799
H	-5.59159377094774	-4.30294328275104	0.93478702073583
C	-6.47699260374100	-2.50894988865293	0.06583839477159
C	-7.81782356955199	-2.62426036214283	0.70619912832792
O	-8.69174905126125	-1.78505980370616	0.61284005422878
O	-7.96359458730439	-3.75615114515355	1.41332706270219
C	-9.19385189124206	-3.93352377973866	2.11639284593606
H	-9.34356894632578	-3.13795016706042	2.86846141227283
H	-9.12250190339348	-4.90721101532475	2.62527304719811
H	-10.05618896141144	-3.94330842829301	1.42617943299539
H	-1.02690218521032	0.72277373582446	-0.35764349290712
H	-2.73148201537193	2.10712237648081	1.22324199614190
H	-4.32617756245934	1.35534087099050	0.94179120967114
C	-6.35817531069899	-1.49799252936905	-1.03839929865829
H	-5.30163208760104	-1.24908242908968	-1.23868895513874
H	-6.77717458609593	-1.91976695957660	-1.97650660104475
H	-6.91089866685481	-0.57058948566566	-0.81870951337376
C	-1.01728258458215	-3.91294858168337	3.50284746732122
H	-0.08354966217254	-3.90108874690174	4.09613741572433
H	-1.39994210956877	-4.94660908228162	3.48632256155816
H	-0.77793398230074	-3.61531903854508	2.46937293256824
CTA-NVP CTI			
C	-0.48256246653091	-0.70693838220045	0.58079329158212
C	-1.61353149722555	0.05188450411667	0.81918661092123
C	-1.82363570238248	1.53310069729390	0.79095588277885
C	-0.88166578574455	-2.06179556309427	0.75612945645180
H	-2.36043969808985	1.88508076425567	1.68990467875695
N	-2.16321376977232	-2.13154197257528	1.09019136095475
N	-2.60158075196990	-0.84919820991166	1.12633399507492
C	-3.97251825952167	-0.57170722914850	1.39261117772261
S	-4.98015900920341	-0.02215178378586	0.22642075937419
S	-4.61135231982333	-0.86923768236709	2.99563622766554
C	-3.18778901869503	-1.37119154074129	4.09702207073721
C	-2.91849059542653	-0.28849978982731	5.14344176951078
H	-2.61572940977062	0.64945864740473	4.64715882764376
H	-3.79950787439632	-0.08971202713025	5.77401609157896
H	-2.08706155628456	-0.61498735466057	5.79459905791356
C	-0.04181372987222	-3.29788393938485	0.62735077371923
H	-0.67117845087785	-4.20306703943105	0.65278752427613
H	0.52718592001926	-3.29585145587347	-0.31869317599427

H	0.69240919105398	-3.36620471570813	1.45067015903252
H	-2.30645604518905	-1.46309763793797	3.44372700153976
H	-4.83174702029307	-2.64211228842937	-2.14331003860720
C	-5.10798340980671	-3.18060235393309	-1.22062214673224
H	-5.34828146060849	-4.22617188074963	-1.50424397798356
H	-4.21898661605088	-3.22521998037517	-0.56713023766503
C	-6.26214109899516	-2.48202996499912	-0.57992747453051
H	-6.93173532425096	-1.87471012782034	-1.20186378775347
H	0.50950048751627	-0.33122302185827	0.31830096089699
H	-0.84829383323152	2.04357997193907	0.74964137805688
H	-2.40922245492251	1.84336046522061	-0.09269716387148
C	-2.68147457098075	-3.86361216037178	4.23871301796080
N	-3.45402940187684	-2.68313321281860	4.63905321868221
C	-4.48196638942096	-2.96183934529830	5.50146790897860
C	-4.39760014276141	-4.44404637670847	5.84827017463531
C	-3.55430009191338	-5.04004217965098	4.71345886348591
H	-2.94962410088435	-5.90830537607907	5.02339573790398
O	-5.30016538234899	-2.15193469496105	5.90758418178469
H	-1.68464188434612	-3.85489381644553	4.72396277701035
H	-2.52610083041546	-3.86461050252695	3.14523840901312
H	-4.21066344650611	-5.36485894223433	3.88599906452787
H	-5.40505695018172	-4.88050003160965	5.95236258527636
H	-3.89713131927421	-4.53177491572267	6.83296480665211
H	-7.62662298794820	-1.82452252259229	3.21866150884437
H	-9.17455136201671	-2.65607164009443	2.93280040564588
C	-8.12872618921331	-2.58277925343558	2.59170844180250
H	-7.93643251672590	-1.09699151018808	0.96350492316040
H	-8.91471472939295	-2.53074741929339	0.52927348768753
C	-8.03583666391270	-2.18699801538407	1.11004198602151
N	-6.82503631330643	-2.86769827542600	0.62615387378620
C	-6.40680518267042	-3.87312208632383	1.48382369478007
O	-5.46074116515994	-4.62170372561306	1.30444876141860
C	-7.36915578228207	-3.91019491686901	2.66382934878786
H	-6.81505220113795	-4.06705854033038	3.60510075234047
H	-8.03402283497718	-4.78612764831017	2.52859701676228
CTA-f CTI			
C	-1.76644731426435	-0.49643250572142	-0.04662096164062
C	-2.72673941755117	-0.34218600645331	0.93618229733415
C	-3.35693317602858	0.89694498095970	1.48256143386462
C	-1.52167567459500	-1.89163082841934	-0.17864065561204
H	-3.52356396959966	0.82655076207797	2.57061610360828
N	-2.28852582191440	-2.55404215734296	0.67150604859906
N	-3.01413169075913	-1.62392145728974	1.34171985632419
C	-3.97523704063781	-2.02735683029345	2.29827458547785
S	-5.64865797845261	-1.58782104552976	2.11085071893030
S	-3.60285540053658	-3.22222355651049	3.49726498643220
C	-2.06601110220156	-2.63342544950724	4.41248762351832
C	-2.10720271165301	-1.12501323631778	4.65826978497627
H	-1.97005162100171	-0.57105222391008	3.71548822504312
H	-3.05294621333189	-0.80194975436441	5.12528448592562
H	-1.27905376383360	-0.83822834672404	5.32994876716680
C	-0.56724648405727	-2.58050179861163	-1.10283243432571
H	-0.62488077725367	-3.67224175593568	-0.96315918466649
H	-0.79714058258276	-2.35105092941771	-2.15861508022900
H	0.47295570236162	-2.25967267678161	-0.91410259093194

C	-2.18420501877233	-3.38664691323810	5.75478030561298
O	-2.19842472561407	-2.85777148198189	6.83854666673550
O	-2.25658084190981	-4.70610686241352	5.56768215584372
C	-2.37648307399216	-5.53847354589237	6.72889645021283
H	-1.49529660062645	-5.43124582405650	7.38511257831161
H	-3.29012733280091	-5.29499584279777	7.29868839434523
H	-2.43710305952576	-6.57330725965538	6.36014557672042
H	-4.59524417600512	-3.84410665261135	0.28929065033131
C	-5.69403192463095	-3.83067081358995	0.39456779465476
H	-6.11746073779969	-4.32138451883179	-0.50074017735766
H	-5.96833187807327	-4.43237392563014	1.27622206165483
C	-6.20278174662397	-2.38986227597540	0.48412162256156
C	-7.73554929241103	-2.28498474120637	0.63842494561593
O	-8.34891339703094	-1.25696358246210	0.47376211363383
O	-8.30250837139362	-3.43624372554598	0.99292447931531
C	-9.72190249156735	-3.43922999823925	1.19311420310128
H	-10.00837424496303	-2.75099914667260	2.00760209493291
H	-9.99044590483832	-4.46981120118411	1.46984585708282
H	-10.25414689517606	-3.15377070575450	0.26897299764985
H	-2.70008778054180	1.76043924489323	1.28788499748807
H	-4.33234149967437	1.09778251341072	1.00442242380479
C	-5.76327141992052	-1.55331226814665	-0.72420147203797
H	-4.66651891125811	-1.57457634443351	-0.84317570211519
H	-6.20208226220614	-1.97316370436336	-1.64905164918682
H	-6.09005207760234	-0.50457517212512	-0.63683125758894
C	-0.77386507963167	-3.06210158907131	3.69643113674963
H	0.09641915590951	-2.86631957708460	4.35086911533131
H	-0.77849929437072	-4.13417291450910	3.44008861411294
H	-0.63117613327830	-2.48404386494222	2.76863530315884
Cl	-0.99650094577734	0.76636351020411	-0.94149229047079

CTA-g CTI

C	-2.93847454932390	-2.42779046230273	1.80004970385392
S	-4.65159101575129	-2.23143427266643	2.18694844126694
S	-2.08767403069108	-3.83292467342685	2.46183022189681
C	-1.76369237006103	-3.41605361428246	4.25518451467027
C	-0.91830708096743	-2.14598258558980	4.37580651279183
H	0.04645459462124	-2.27539715463455	3.85543965098783
H	-1.42878380091608	-1.26403048314407	3.95247367069791
H	-0.70421221078850	-1.92772317518720	5.43785097189565
C	-3.09500113183356	-3.23652733577592	5.00919631351384
O	-3.42563672995263	-2.21126790452957	5.55653237655579
O	-3.82336713744535	-4.35440916926904	5.03848405477821
C	-5.09147126979762	-4.29007076886900	5.69393632696001
H	-4.97382953284755	-4.07278646705461	6.77084069262143
H	-5.73719596027153	-3.51795103787494	5.24013391199882
H	-5.55557230290203	-5.28072271859887	5.57228051534556
H	-4.76509879636315	-5.27948791573147	2.16157094715102
C	-5.74164434787057	-4.82556604240527	1.92342434512343
H	-6.32330365797280	-5.55203878929705	1.32393643082881
H	-6.28077833075909	-4.65164037572559	2.86809130761389
C	-5.54071301534727	-3.52604668903595	1.12906541014837
C	-6.88701193924077	-2.83520322615331	0.85068796142125
O	-7.27607881314228	-2.49283977473799	-0.24003000255600
O	-7.58212705018290	-2.64294489203433	1.97647606595957
C	-8.83984875183424	-1.96663187254377	1.87276758271111

H	-8.71217613268963	-0.95001834631436	1.46088690946603
H	-9.23974523431282	-1.90070719332212	2.89597030980543
H	-9.54498579810402	-2.53113277802822	1.23778334875655
C	-4.80840324107190	-3.78469005284928	-0.18758756605240
H	-3.83560611185821	-4.27284749945985	-0.00684140964746
H	-5.40804913832918	-4.46727683558545	-0.81648221523441
H	-4.63396641824715	-2.86064225406165	-0.76351041316234
C	-1.00592423642700	-4.63939469784973	4.80645282773231
H	-0.81100327496368	-4.50583758226120	5.88652514573265
H	-1.57398062828942	-5.57571252364670	4.67583900062618
H	-0.03045210766258	-4.74904545458536	4.29991376135792
C	-2.26260727207361	-1.48995050539774	0.94498122944509
C	-2.95311945037729	-0.44088188585064	0.26412549664705
C	-0.85391749451425	-1.54271941822935	0.71428417952122
C	-2.30819561541784	0.46477926879852	-0.55731657377241
C	-0.19936391609518	-0.64289474921330	-0.10578619612116
C	-0.90044095109432	0.40233763034135	-0.77637399872209
H	-4.03708124221011	-0.34672404854780	0.39372347974481
H	-0.26369432254730	-2.32683869712860	1.20117215112887
H	-2.91092441974844	1.23774818615491	-1.04478167728451
H	0.88294272090001	-0.75166153949656	-0.22842134703427
N	-0.25722451950418	1.29589831087974	-1.58773196445551
C	-1.00378174678906	2.35208857172022	-2.25434791849021
H	-1.51495360614255	3.01856565077921	-1.53243227526514
H	-0.31125330909080	2.97091738734941	-2.84456948897345
H	-1.76801002306471	1.94912670124208	-2.94784799665434
C	1.17824059433583	1.19209435095300	-1.80176185346458
H	1.46199841055360	0.22424908000613	-2.25983085563757
H	1.50326994839871	1.99134197257186	-2.48462052998944
H	1.74899787703985	1.30431034512268	-0.85911899210472

CTA-MA TS

C	-1.65230394630010	-0.62108313900502	0.59107843219712
C	-2.61175840269151	-0.47783824583906	1.56811196440646
C	-1.76898951710909	-1.96316259200524	0.11887957756858
H	-0.93896667165366	0.14042334244628	0.26620455762714
C	-2.91050558655080	0.70548587193073	2.43390397935905
N	-3.24853726281084	-1.70422364878233	1.64508259103913
N	-2.72492319865221	-2.60444901628566	0.76586512273020
C	-0.96481120030047	-2.63640368388649	-0.95130430099068
H	-3.02151819423754	0.41853379663430	3.49381423199913
H	-2.07736063661409	1.42285131971642	2.35766858840725
H	-3.83533871183108	1.22142440177291	2.12384333263588
C	-4.34765985753253	-2.07742392371123	2.44023343552628
H	-1.29178710106279	-3.68101396331521	-1.08277530828736
H	-1.07226583087246	-2.11163476889887	-1.91728615815061
H	0.11020050143375	-2.63548138043367	-0.69715178776810
S	-5.67430820687127	-1.11589310796373	2.64071773027735
S	-4.38271686805429	-3.70389065542143	3.10317114352497
H	-2.00054064862071	-3.83813877766448	2.67394817845077
C	-2.63461359696829	-4.11511318081295	3.53116354141886
C	-2.22000309366128	-3.24468245837818	4.71575640759975
C	-2.55549596155453	-5.62247989597925	3.78037212021088
O	-1.59950790606678	-2.21347393659328	4.60266040684515
O	-2.64359638969939	-3.73544669200761	5.88024831358666
H	-1.53039853943308	-5.89412132384545	4.08846752900831

H	-3.24876447096297	-5.95643616387276	4.57135785943886
H	-2.78648872811710	-6.17533920605798	2.85358152886132
C	-2.35775341783483	-2.97631996610213	7.06159213362408
H	-1.27001018458065	-2.84931452494670	7.20061546586527
H	-2.83933466312086	-1.98344090214422	7.01995663546458
H	-2.77174974961085	-3.55048316702467	7.90412911846829
H	-7.75044202963394	-0.65011732913044	0.78825093131355
C	-7.06236639820079	-1.47085102489953	0.54314829459128
C	-6.07929653086481	-1.26499472203383	-0.56343410435237
C	-7.58422305041794	-2.80182019868909	0.90880943306623
H	-5.66208365248895	-0.24387822426702	-0.54038411783813
H	-6.58397742303305	-1.38872708907327	-1.54463392423192
H	-5.25103766053759	-1.99334361377887	-0.53329574191695
O	-8.61278691783985	-2.98845830525144	1.52844701929817
O	-6.78160659957999	-3.79756936485644	0.49184572997139
C	-7.14603693438610	-5.13023100727106	0.84909925431907
H	-7.27603616022415	-5.23720191965137	1.94074128198891
H	-6.32159310116103	-5.77852330914741	0.51399151831328
H	-8.08056449968948	-5.43845930347324	0.34602005453290
CTA-DMA TS			
C	-1.09512488258070	-1.29759473144753	0.18172348685014
C	-2.11067626035369	-0.54514793135289	0.73063664161053
C	-1.39035828520725	-2.65412493214992	0.50877045195449
H	-0.23507181921684	-0.91809987934056	-0.37556590290039
C	-2.29464586518937	0.94018163741301	0.73271199511067
N	-2.94985549160111	-1.45149004035281	1.35279042992062
N	-2.50206372414900	-2.72964220237347	1.21781999937592
C	-0.60849756038003	-3.88081180438953	0.14826307820052
H	-2.56556099397018	1.32038046478416	1.73343099812884
H	-1.34941689866068	1.41822494913136	0.42848155620351
H	-3.08140578662730	1.25847024488928	0.02736964098647
C	-4.16530888384394	-1.20342713296782	2.02510184315257
H	-1.09526656073498	-4.78555892536047	0.54848853193629
H	-0.52084926461167	-3.98759320978996	-0.94759804933650
H	0.41754321392119	-3.82840251994551	0.55415561353878
S	-5.34075772769562	-0.21678557980894	1.41211121439212
S	-4.51277056663282	-2.09816468646557	3.49217455781082
H	-6.44420935653819	-1.82000539584013	-0.70064641970482
C	-7.10317022169429	-1.48624238429711	0.10932170688055
C	-7.52264474977785	-2.45723101319659	1.15870129927231
C	-8.00880162851204	-0.33862028548740	-0.19922088340385
O	-8.47967378253293	-2.19897764814903	1.89068569472926
N	-6.79986805270122	-3.61832787136567	1.27950040155618
H	-7.48689442061123	0.44326687677052	-0.77805970163424
H	-8.85771643490864	-0.68693957794048	-0.82427374545631
H	-8.44181201901325	0.10506253866729	0.71293393828537
C	-7.18814240165354	-4.59893115032210	2.28011407752993
C	-5.76167685213202	-4.04342056140230	0.34934910764234
H	-6.32164949750329	-4.86810326757103	2.91415063587305
H	-7.56970485827052	-5.52614122249622	1.80786931045156
H	-7.97845453500192	-4.18226929865475	2.92029565412812
H	-6.18215685070479	-4.52691119699224	-0.55553972122836
H	-5.11150988778012	-3.21300257288838	0.03306787820416
H	-5.11247253665328	-4.77979142399682	0.85255318410218
H	-2.21392280285087	-2.87204230392386	3.56412328473591

C	-2.88541600645441	-2.42094718995909	4.31208445513538
C	-3.20386402332738	-3.44372117781306	5.43017930438019
C	-2.26794624975112	-1.14130640941479	4.86699980826435
N	-3.34762293494736	-4.75140417999265	5.06714566428258
O	-3.33859099591956	-3.05460939539514	6.58328588358459
H	-2.07352739259612	-0.41912659722596	4.05452383259313
H	-2.92039664300489	-0.66281128438071	5.61559545023278
H	-1.29941692370585	-1.36873955986440	5.34798920583415
C	-3.06624606728811	-5.29611497706007	3.74342122539344
C	-3.61950807993773	-5.74765084242608	6.09502426799792
H	-3.17755424100614	-4.54754477341699	2.94527172948424
H	-2.04201537657540	-5.71619360668038	3.68835778960541
H	-3.77765726630017	-6.11322276017479	3.52985579432220
H	-2.74194505772265	-6.40498635324443	6.25297442711139
H	-3.86091423437786	-5.25072663624448	7.04499867113655
H	-4.47363426071123	-6.38181521809350	5.79408070174292
CTA-MMA TS			
C	-1.55027131188320	-0.10419603190065	0.12878037911870
C	-2.63850088000580	-0.00233559779882	0.96829741254376
C	-3.35255945115319	1.22399378960965	1.44449697848281
C	-1.24761795407107	-1.49565773034754	0.04623786773626
H	-3.53720447407017	1.19716189800645	2.53274374105280
N	-2.08126941082199	-2.19857724441417	0.79229898686517
N	-2.93369344045758	-1.29813388578185	1.35129533079222
C	-3.98211251187646	-1.75215065675546	2.17656098224934
S	-5.53482140553612	-1.20638375569223	2.00103121804723
S	-3.71055930194734	-3.10337674965367	3.26506673240555
C	-2.03156646328082	-2.93228239668915	4.11209772893031
C	-1.50271929256804	-1.49684341725057	4.13168831672944
H	-1.12106712297105	-1.19592767073835	3.14333875993376
H	-2.26359637835301	-0.76926250138202	4.46199371641662
H	-0.65592984458750	-1.43919821997274	4.83790356829774
C	-0.15533824590939	-2.15753054064772	-0.73825570474593
H	-0.21207282107842	-3.25386483844339	-0.63569008704419
H	-0.22536376378181	-1.90284589425010	-1.81061120346626
H	0.83883912301426	-1.82610141230024	-0.38824498356944
C	-2.39512006705152	-3.36417218588213	5.55189174582236
O	-2.30179575276466	-2.65858567173246	6.52550080307301
O	-2.83247361994081	-4.62490298277512	5.58480012560001
C	-3.24764188764546	-5.15347510649752	6.85080611750270
H	-2.41058339159362	-5.16249190770912	7.57042788274912
H	-4.08487284622180	-4.56780185833269	7.26930197900015
H	-3.57946063556648	-6.18495828611719	6.65927924417026
H	-4.53408556112039	-3.41276656810120	-0.20905591519375
C	-5.57611992393995	-3.70780130542640	0.00829514693719
H	-5.89922232472086	-4.37531652838319	-0.81802399146799
H	-5.59159377094774	-4.30294328275104	0.93478702073583
C	-6.47699260374100	-2.50894988865293	0.06583839477159
C	-7.81782356955199	-2.62426036214283	0.70619912832792
O	-8.69174905126125	-1.78505980370616	0.61284005422878
O	-7.96359458730439	-3.75615114515355	1.41332706270219
C	-9.19385189124206	-3.93352377973866	2.11639284593606
H	-9.34356894632578	-3.13795016706042	2.86846141227283
H	-9.12250190339348	-4.90721101532475	2.62527304719811
H	-10.05618896141144	-3.94330842829301	1.42617943299539

H	-1.02690218521032	0.72277373582446	-0.35764349290712
H	-2.73148201537193	2.10712237648081	1.22324199614190
H	-4.32617756245934	1.35534087099050	0.94179120967114
C	-6.35817531069899	-1.49799252936905	-1.03839929865829
H	-5.30163208760104	-1.24908242908968	-1.23868895513874
H	-6.77717458609593	-1.91976695957660	-1.97650660104475
H	-6.91089866685481	-0.57058948566566	-0.81870951337376
C	-1.01728258458215	-3.91294858168337	3.50284746732122
H	-0.08354966217254	-3.90108874690174	4.09613741572433
H	-1.39994210956877	-4.94660908228162	3.48632256155816
H	-0.77793398230074	-3.61531903854508	2.46937293256824

CTA-NVP TS

C	-0.48256246653091	-0.70693838220045	0.58079329158212
C	-1.61353149722555	0.05188450411667	0.81918661092123
C	-1.82363570238248	1.53310069729390	0.79095588277885
C	-0.88166578574455	-2.06179556309427	0.75612945645180
H	-2.36043969808985	1.88508076425567	1.68990467875695
N	-2.16321376977232	-2.13154197257528	1.09019136095475
N	-2.60158075196990	-0.84919820991166	1.12633399507492
C	-3.97251825952167	-0.57170722914850	1.39261117772261
S	-4.98015900920341	-0.02215178378586	0.22642075937419
S	-4.61135231982333	-0.86923768236709	2.99563622766554
C	-3.18778901869503	-1.37119154074129	4.09702207073721
C	-2.91849059542653	-0.28849978982731	5.14344176951078
H	-2.61572940977062	0.64945864740473	4.64715882764376
H	-3.79950787439632	-0.08971202713025	5.77401609157896
H	-2.08706155628456	-0.61498735466057	5.79459905791356
C	-0.04181372987222	-3.29788393938485	0.62735077371923
H	-0.67117845087785	-4.20306703943105	0.65278752427613
H	0.52718592001926	-3.29585145587347	-0.31869317599427
H	0.69240919105398	-3.36620471570813	1.45067015903252
H	-2.30645604518905	-1.46309763793797	3.44372700153976
H	-4.83174702029307	-2.64211228842937	-2.14331003860720
C	-5.10798340980671	-3.18060235393309	-1.22062214673224
H	-5.34828146060849	-4.22617188074963	-1.50424397798356
H	-4.21898661605088	-3.22521998037517	-0.56713023766503
C	-6.26214109899516	-2.48202996499912	-0.57992747453051
H	-6.93173532425096	-1.87471012782034	-1.20186378775347
H	0.50950048751627	-0.33122302185827	0.31830096089699
H	-0.84829383323152	2.04357997193907	0.74964137805688
H	-2.40922245492251	1.84336046522061	-0.09269716387148
C	-2.68147457098075	-3.86361216037178	4.23871301796080
N	-3.45402940187684	-2.68313321281860	4.63905321868221
C	-4.48196638942096	-2.96183934529830	5.50146790897860
C	-4.39760014276141	-4.44404637670847	5.84827017463531
C	-3.55430009191338	-5.04004217965098	4.71345886348591
H	-2.94962410088435	-5.90830537607907	5.02339573790398
O	-5.30016538234899	-2.15193469496105	5.90758418178469
H	-1.68464188434612	-3.85489381644553	4.72396277701035
H	-2.52610083041546	-3.86461050252695	3.14523840901312
H	-4.21066344650611	-5.36485894223433	3.88599906452787
H	-5.40505695018172	-4.88050003160965	5.95236258527636
H	-3.89713131927421	-4.53177491572267	6.83296480665211
H	-7.62662298794820	-1.82452252259229	3.21866150884437
H	-9.17455136201671	-2.65607164009443	2.93280040564588

C	-8.12872618921331	-2.58277925343558	2.59170844180250
H	-7.93643251672590	-1.09699151018808	0.96350492316040
H	-8.91471472939295	-2.53074741929339	0.52927348768753
C	-8.03583666391270	-2.18699801538407	1.11004198602151
N	-6.82503631330643	-2.86769827542600	0.62615387378620
C	-6.40680518267042	-3.87312208632383	1.48382369478007
O	-5.46074116515994	-4.62170372561306	1.30444876141860
C	-7.36915578228207	-3.91019491686901	2.66382934878786
H	-6.81505220113795	-4.06705854033038	3.60510075234047
H	-8.03402283497718	-4.78612764831017	2.52859701676228

CTA-f TS

C	-1.57589059677113	-0.11386412178100	0.13735063312078
C	-2.65218091904265	0.00266380960193	0.99426838301718
C	-3.34803841743655	1.22911723855311	1.48709860289064
C	-1.26608223158261	-1.50079999572225	0.03216581407587
H	-3.56321498729229	1.16901323753414	2.56755060770369
N	-2.09723289343146	-2.19349692021393	0.78804561013386
N	-2.93567125511331	-1.29492604698354	1.36402521288345
C	-3.98553095193139	-1.75471863555209	2.19116336362764
S	-5.53550250909713	-1.21147478476482	2.00912309950630
S	-3.70346377371275	-3.10545433013541	3.27441549902558
C	-2.02420423341433	-2.92226524185017	4.11905550543141
C	-1.50847750110336	-1.48225905968814	4.14444221837406
H	-1.12734123993238	-1.17360330865043	3.15827181639624
H	-2.27584947664353	-0.76267723910043	4.47703335896256
H	-0.66296714384468	-1.42031268982423	4.85165012771319
C	-0.18514919640889	-2.14703011077962	-0.77473236220789
H	-0.23634635560419	-3.24319596059342	-0.67325812761223
H	-0.27853997897480	-1.89001389991377	-1.84479381442989
H	0.81298300761395	-1.81173766432028	-0.44014140357127
C	-2.38130077510632	-3.36468125926889	5.55734388882850
O	-2.28709749651921	-2.66485532876303	6.53481687174698
O	-2.81337448767259	-4.62712717288609	5.58295810708516
C	-3.22148981689911	-5.16661358913778	6.84685459601965
H	-2.38132907018601	-5.17759374887647	7.56277759387276
H	-4.05919174170987	-4.58710412199113	7.27284550207240
H	-3.55012442539968	-6.19786838191032	6.64894988465304
H	-4.53220628986304	-3.46408634426134	-0.17374366672192
C	-5.58037164647900	-3.74324808172087	0.03454567323652
H	-5.90011931312065	-4.42151072772521	-0.78438380515091
H	-5.61681387052664	-4.32226807824875	0.97063427227004
C	-6.46769158333025	-2.53395890853094	0.05756838061807
C	-7.81783307757866	-2.61915572337138	0.68159794252829
O	-8.67667397810302	-1.76732015540062	0.56568586728919
O	-7.99056861173235	-3.73937463219414	1.40076816927016
C	-9.23362596595324	-3.88975393673995	2.08756433660924
H	-9.38240744761156	-3.08274699017031	2.82748972622809
H	-9.18428414638240	-4.85790470555818	2.60934686792311
H	-10.08556014670672	-3.89542058427474	1.38450954812682
H	-2.70196901918221	2.10495421267307	1.31331255036259
H	-4.30213549248102	1.39795113340899	0.95861764232121
C	-6.31318859369871	-1.53514506687324	-1.05236329909979
H	-5.24962897964693	-1.30485285277823	-1.23706425528790
H	-6.72248053741072	-1.95927058762346	-1.99375124780320
H	-6.85514561365249	-0.59743051092835	-0.85067261365845

C	-1.00247329156767	-3.89142236604020	3.50428905250532
H	-0.06964709764808	-3.87540151941057	4.09873386463389
H	-1.37720321208721	-4.92779115824175	3.48174821680667
H	-0.76312968533793	-3.58688815371583	2.47291436538459
Cl	-0.74057947268286	1.17641531474426	-0.65023072771147

CTA-g TS

C	-3.03578472436909	-2.61577689139131	1.45088978640497
S	-4.77221222544479	-2.34293732283256	1.78036524324426
S	-2.27881813140941	-3.98047903565325	2.05201386252110
C	-1.53217588870721	-3.51286914272831	4.42026624249445
C	-0.36899330446410	-2.58180537853028	4.23610399652053
H	0.44710662802172	-3.06318366363230	3.67076653538431
H	-0.65773557046106	-1.64336266199763	3.73460762713913
H	0.04644718008044	-2.30258751717232	5.22812082596408
C	-2.79773192792341	-2.87131532456471	4.86710353375299
O	-2.98197305512312	-1.66978764747095	4.87166183941256
O	-3.72147023453997	-3.75371445904225	5.28093294380571
C	-4.97996746331838	-3.22771528083377	5.69996978642098
H	-4.86783834311328	-2.57079829495930	6.58168426541511
H	-5.46835922525096	-2.65866147546249	4.88981155826797
H	-5.60408873140584	-4.09360270165581	5.97096718904326
H	-4.76115095539533	-5.42614039827525	2.17935319906238
C	-5.76296607733592	-4.99861216259178	2.01012924937051
H	-6.40947785936360	-5.78970297471648	1.58472230528388
H	-6.17879628020164	-4.70110899687264	2.98657304096941
C	-5.67717179689523	-3.81365497267088	1.03442665754876
C	-7.08143479873923	-3.21393216884790	0.80465826350567
O	-7.61222955272255	-3.09663867256708	-0.27256352398220
O	-7.64699219329818	-2.83720697896551	1.95460523001552
C	-8.94584207108985	-2.23601153843639	1.89130855873168
H	-8.92594429343715	-1.30962354038516	1.29080198425973
H	-9.22453899957451	-1.99594099643907	2.92843030293883
H	-9.68638859940243	-2.93355152005886	1.46247925301797
C	-5.07129586462901	-4.22613140445882	-0.30839076426137
H	-4.06770971633786	-4.66100225114799	-0.16981132757453
H	-5.70444127469478	-4.99887630885300	-0.77986371138345
H	-4.99081317613514	-3.37677576254915	-1.00759801276082
C	-1.23127772660006	-4.92827942402302	4.82600749678216
H	-0.91123987928475	-4.95823001932260	5.88872256451358
H	-2.10339853985057	-5.59434085467873	4.72902206032525
H	-0.39932861922709	-5.34120616320445	4.22828167194394
C	-2.33176332424607	-1.54467422756544	0.74108612691515
C	-3.00480939274893	-0.48311663244364	0.08481218811507
C	-0.91537874165300	-1.53074579475745	0.65157496827282
C	-2.32835814723074	0.51847035254871	-0.59261529871155
C	-0.22281985423936	-0.53947422017947	-0.01883671227681
C	-0.90625337506270	0.53297931433935	-0.66552840569718
H	-4.09969452482981	-0.44953719400345	0.09612755394218
H	-0.34433961141160	-2.33634691393623	1.12545088421213
H	-2.91837902496455	1.30003821864570	-1.08109298955440
H	0.86990087787245	-0.59521489476121	-0.04071021530927
N	-0.23020769432408	1.51888615417405	-1.32398110677178
C	-0.95686339863984	2.60492910951138	-1.96538031596796
H	-1.57282219992492	3.17427941293698	-1.24237762113346
H	-0.23959156809598	3.30684511252729	-2.41653415357006

H	-1.62366632239175	2.24133476066379	-2.77184858405783
C	1.22325384662024	1.48989174460937	-1.39961445980727
H	1.59490579820971	0.57683771441385	-1.90435710193061
H	1.57477008975278	2.35641537312780	-1.97954319959613
H	1.69347585895152	1.54355951714063	-0.39824629117139

MA radical

H	-7.80117575705961	-0.66256519575926	0.74307809260823
C	-7.17578857175054	-1.49829490415563	0.40328600993500
C	-6.06972043268246	-1.25392834636416	-0.55890832701235
C	-7.59892111517409	-2.80892833841497	0.88121963323285
H	-5.65626819048805	-0.23758754576166	-0.43904104436864
H	-6.43856589139860	-1.32398936806110	-1.60524058586420
H	-5.25424247142259	-1.99069044373638	-0.45996697881943
O	-8.55581536407892	-2.98882246058343	1.61563018810519
O	-6.82179590649705	-3.81462504470255	0.42932592249311
C	-7.15143188584191	-5.13616266537835	0.85395051889809
H	-7.13600295793938	-5.22439113970409	1.95526055774829
H	-6.38538891707113	-5.80116899877003	0.42546888427934
H	-8.14693348859559	-5.44302094860837	0.48454274876451

DMA radical

H	-6.56194182441991	-1.91936958477181	-0.79928035820085
C	-7.25644004359965	-1.60473028509413	-0.01202128583650
C	-7.56320371926881	-2.48748918292494	1.12504556939234
C	-8.00042609028528	-0.33105235263117	-0.19968592518001
O	-8.47752603352215	-2.20044332648379	1.90614527219909
N	-6.80945688408772	-3.63027707574712	1.27568629014095
H	-7.38511445168412	0.41892449679048	-0.72800392185504
H	-8.90463286317271	-0.48954205512962	-0.82704201092406
H	-8.34997415048237	0.08768384924824	0.75923425176163
C	-7.18126124298746	-4.60564175790543	2.28778962659562
C	-5.73772675674172	-4.02141740956057	0.37017292246839
H	-6.32057346769446	-4.84137132673824	2.94246261423728
H	-7.52649666924069	-5.55218097191914	1.82526334647431
H	-7.99463609072335	-4.20245715100648	2.90767001091306
H	-6.11371288614612	-4.50204271335345	-0.55566814937251
H	-5.10137869470945	-3.16577587581481	0.09060695898107
H	-5.08605622123395	-4.74940272695799	0.88242720820524

MMA radical

H	-4.56761269331405	-3.38035627279866	-0.23186989443498
C	-5.59186547845790	-3.73941960383491	-0.02682944726463
H	-5.82269109607234	-4.50009314561010	-0.80346267299232
H	-5.59651561107662	-4.26075270087678	0.94442318288521
C	-6.57434313861964	-2.61284766648836	-0.08034193470897
C	-7.84822960245165	-2.66701862595119	0.64218864588661
O	-8.71208262757006	-1.80667357245038	0.58696060942751
O	-7.98224729747929	-3.78022313224834	1.39442090881754
C	-9.18508848764793	-3.91620157239787	2.14789376489208
H	-9.30075227393753	-3.09589301659782	2.87952541534030
H	-9.10870080061060	-4.87388432323456	2.68633485563184
H	-10.07467518150267	-3.93713536622152	1.49260057028131
C	-6.35624660577490	-1.48387419084565	-1.03439682427194
H	-5.28953504419296	-1.19948995800295	-1.08236308672559
H	-6.64803351126981	-1.78142420469229	-2.06482135822342
H	-6.95655429002197	-0.59738634774858	-0.77205347454057

NVP radical

H	-4.82183873043067	-2.62291095178977	-2.13646883628045
C	-5.11296974275400	-3.19072188369798	-1.23568562610492
H	-5.35432184734456	-4.22373111080462	-1.56570369258968
H	-4.22680239469611	-3.27348156908128	-0.58155637115304
C	-6.26453318994978	-2.50052232015421	-0.58279739867307
H	-6.88728404218827	-1.80869078604518	-1.16265773247495
H	-7.62294752651691	-1.81315872830027	3.20771029300519
H	-9.17389592713899	-2.64641348889572	2.94076455410971
C	-8.12965221632139	-2.57930483574889	2.59317956594111
H	-7.96818727269863	-1.11214205544565	0.94389208080047
H	-8.91891375506707	-2.56825167355844	0.53265924896235
C	-8.04453089400938	-2.20156624625464	1.10680024972478
N	-6.82264595552955	-2.87056870479320	0.63426577868503
C	-6.40256863189737	-3.86702285195840	1.49611071108754
O	-5.43968329296139	-4.59886415843114	1.33019168906395
C	-7.37291410562778	-3.90860239792247	2.67206588912104
H	-6.82382265227712	-4.06733949704817	3.61590326201729
H	-8.04106067259097	-4.78156890006996	2.53217286475765
CTA-MA			
C	-1.67621997383047	-0.57568587122532	0.63172979036601
C	-2.67875616445028	-0.41754402839039	1.55641187878616
C	-1.72223131158566	-1.94619862136210	0.22381972786424
H	-0.97845393667888	0.19494413765043	0.29506279521107
C	-3.04917270078746	0.80307068424852	2.33911542919152
N	-3.27101769387071	-1.67114862520188	1.67622222181431
N	-2.67406947073540	-2.59256584559644	0.86376329814695
C	-0.84996848929584	-2.63352855511331	-0.78080560711302
H	-3.19411129938565	0.57961094327318	3.41011909512738
H	-2.23471927143545	1.53993580241601	2.24859302280051
H	-3.97607634165492	1.26822043811096	1.96333339368921
C	-4.37972969155348	-2.05065272796360	2.43917986649379
H	-1.12767262589023	-3.69616521768697	-0.87433693416383
H	-0.94042982639442	-2.15754960379988	-1.77335424535881
H	0.21240265955075	-2.57237650839674	-0.48471946468261
S	-5.70429223972766	-1.10787359076498	2.63851075922586
S	-4.41690900072907	-3.66767629229723	3.12834897452623
H	-2.06266220017087	-3.95737501831501	2.61179404776373
C	-2.67894658076981	-4.17611914090560	3.49703496425796
C	-2.16265339639638	-3.31566684109508	4.64899661990839
C	-2.69432826133821	-5.68042842878757	3.77693235948216
O	-1.47308296883868	-2.33573454923971	4.49289518558882
O	-2.57844105430745	-3.75426191426580	5.83660347869490
H	-1.67867956575268	-6.01411433398012	4.05371504994674
H	-3.37766872943032	-5.95102868232227	4.60012089552782
H	-2.99552024302753	-6.23523793735997	2.87173799640834
C	-2.19374358912049	-2.99991182802229	6.99287085625905
H	-1.09519785657447	-2.95489829051278	7.09187776240437
H	-2.59938466494025	-1.97380748236716	6.94858896789529
H	-2.62007156087800	-3.52680167072691	7.85974418393741
CTA-DMA			
C	-1.11320461078009	-1.24795591514717	0.15664427242760
C	-2.14066028387020	-0.50245570295313	0.68208926796143
C	-1.35464157464578	-2.59904627581291	0.55765728953026
H	-0.27646220148778	-0.87004512400877	-0.43588214242066
C	-2.36535004748978	0.97495108277090	0.60641572089098

N	-2.93589148710441	-1.40799422209270	1.37573992171903
N	-2.44340942491839	-2.67814221807557	1.29419906525136
C	-0.53809999601439	-3.81246312737890	0.23607033984086
H	-2.62634328663640	1.40473755196074	1.58906123262661
H	-1.43736216026298	1.45540039817764	0.25644880896991
H	-3.17276447372102	1.23252519712524	-0.09969380688042
C	-4.14622301353810	-1.18744612421618	2.04649788130156
H	-0.98028614696903	-4.71284750655215	0.69335270179904
H	-0.47410443162037	-3.96769232770992	-0.85550786200917
H	0.49467269781673	-3.69985921921897	0.61118175698745
S	-5.32128394629830	-0.19843192318477	1.47588824331559
S	-4.46971839583875	-2.08749346646715	3.51597715585450
H	-2.19648615689018	-2.94287953208254	3.56181548279382
C	-2.84285660755864	-2.47032038548592	4.31710836996506
C	-3.18493564092709	-3.48167945638267	5.43922552806998
C	-2.18008044562973	-1.21117073363504	4.86580498674497
N	-3.38058520106503	-4.78145572262041	5.07556316465336
O	-3.28671275165290	-3.08930867780580	6.59441193920017
H	-1.96693954573705	-0.49589410634601	4.05207237922937
H	-2.80918535650197	-0.71114133110788	5.62026841011743
H	-1.21628856318696	-1.47250100431916	5.33869343796154
C	-3.14743232820399	-5.33189480113788	3.74475067554245
C	-3.67026500888468	-5.77072020219651	6.10555184161463
H	-3.24629083583196	-4.57862411848607	2.94911007727901
H	-2.14215701516069	-5.79183686566729	3.66768460250008
H	-3.89631612225493	-6.11804947949348	3.54389332798383
H	-2.81493310913436	-6.46078674721487	6.24408314085416
H	-3.87455175198823	-5.26868217098605	7.06151479952719
H	-4.55311668601255	-6.37134529224862	5.81908556879702

CTA-MMA

C	-1.58797101825442	-0.08732027924819	0.12992431623390
C	-2.68928151378749	0.02714852149777	0.94229087566589
C	-3.43095652050116	1.26205950174963	1.34782071249681
C	-1.24314549268146	-1.47572178641381	0.11882611992136
H	-3.61979682975140	1.29106605248478	2.43507449664282
N	-2.05930833800311	-2.16547699237766	0.88822105301252
N	-2.95111451971672	-1.26336330943294	1.39188386891828
C	-3.99538060363487	-1.71731322589214	2.20200237881331
S	-5.52782952491685	-1.14617995058110	2.08847466550867
S	-3.71622337128142	-3.06704091786709	3.29249792096814
C	-2.00598625890513	-2.95938883412939	4.09177602543445
C	-1.43068474508834	-1.54166802290848	4.11003274308771
H	-1.05394859327171	-1.24611785131834	3.11872371421981
H	-2.16167288483222	-0.79151191064208	4.45727948861101
H	-0.57198863189522	-1.51763849270194	4.80351969246199
C	-0.12419971596231	-2.13512056966084	-0.62717456851440
H	-0.15120193880236	-3.22831574453504	-0.48844270966455
H	-0.18814266644906	-1.91711390721802	-1.70780331734856
H	0.85431706183754	-1.76161812151762	-0.27569377702505
C	-2.35713355427374	-3.38620303567111	5.53836671379071
O	-2.20710260509246	-2.69370562507236	6.51372077087108
O	-2.85023758484491	-4.62545908911146	5.57143156813591
C	-3.25509583395630	-5.14870004443327	6.84357634800446
H	-2.39985567451668	-5.20326640961497	7.53930762694550
H	-4.05301086645915	-4.52988879996812	7.29001926670880

H	-3.63858092104711	-6.16180284221548	6.65087601563708
H	-1.07883884996147	0.72843686241677	-0.38915726673115
H	-2.82232372282168	2.14124807894682	1.08104639760467
H	-4.40320937800950	1.34770056114414	0.83398289360048
C	-1.03765578661185	-3.97471081156134	3.46652531889341
H	-0.09864437922074	-3.99826317972023	4.05083329026731
H	-1.45834608510016	-4.99346587066856	3.45270156061565
H	-0.79809189218653	-3.68191328375831	2.43264355621200

CTA-NVP

C	-0.66440579997813	-1.00734792802488	0.39593010458972
C	-1.55471360703772	0.00463031101203	0.66222702540116
C	-1.39894177945575	1.47623139492083	0.43750423672782
C	-1.28522932424121	-2.21289654335077	0.84959401785516
H	-1.72119003218591	2.06382114354515	1.31446357241272
N	-2.46480886751305	-1.96139034818399	1.37960889047907
N	-2.64447631120866	-0.61184482014089	1.26499198635728
C	-3.87558669315058	-0.05661065788330	1.64284370963611
S	-4.65167538356004	1.08186832589216	0.75129545463385
S	-4.68022918867528	-0.68899898158297	3.06264246090616
C	-3.33800697577677	-1.21852031135182	4.24542438303155
C	-3.42455327444115	-0.34707667129942	5.50278205287672
H	-3.26474896723406	0.71538097653539	5.25159656650411
H	-4.40031216013404	-0.44809612532658	6.00723278117523
H	-2.63286655849511	-0.65369670155824	6.21080387742611
C	-0.73735511230515	-3.60573902726396	0.77843079500687
H	-1.49536320428470	-4.34176378623451	1.09278959590883
H	-0.41810276517778	-3.85045701852794	-0.24992544186417
H	0.14924744064046	-3.71501526245443	1.42881047853485
H	-2.37909174462096	-1.02814591316003	3.73731848510153
H	0.32338292986291	-0.89814305718183	-0.05853117166677
H	-0.33512626276159	1.69394841246466	0.24884647368802
H	-1.98036761259848	1.82386951262229	-0.43308034044769
C	-2.22821801526368	-3.49314002882050	4.57083044741449
N	-3.41442276802822	-2.63592012882703	4.51895377119904
C	-4.54606501090792	-3.24205615023464	4.99155045812612
C	-4.18980197939471	-4.68214824085446	5.33971766488023
C	-2.83123788770892	-4.90576143199522	4.66179929983974
H	-2.17561232222520	-5.60329281293549	5.20865587830743
O	-5.63038399030262	-2.69385813003524	5.11572430189849
H	-1.60893192790699	-3.24983559020881	5.45878818144891
H	-1.60223383339891	-3.34907453366285	3.67373019172610
H	-2.97881853950067	-5.31261886884750	3.64468924766775
H	-4.98629918775347	-5.37335594187412	5.01642681590488
H	-4.12331442327551	-4.75032091517079	6.44349321731206

CTA-f

C	-2.13523128441859	-0.14261606551785	0.20173847837275
C	-3.07643774474899	-0.00102310774984	1.19629192809219
C	-3.71702322229563	1.24132924331752	1.72288406445860
C	-1.79575465522304	-1.52938425930851	0.12625112742404
H	-3.75190991302253	1.24451439641488	2.82549234269285
N	-2.47444617148981	-2.19747205933411	1.03357562793248
N	-3.25394093006423	-1.28757092353317	1.68041101381069
C	-4.15460085052939	-1.73699294708639	2.65742283119184
S	-5.68474827209077	-1.16809674363954	2.77892352005598
S	-3.69099665916828	-3.07145979329752	3.69835185632776

C	-1.87065211390583	-2.94860695112681	4.19693557764380
C	-1.30855854908414	-1.52824966794501	4.10857224837430
H	-1.09457659802525	-1.24179396480297	3.06723437347628
H	-1.97851883918276	-0.77688440668253	4.56064415574284
H	-0.35041392869119	-1.49338175524420	4.65562734275841
C	-0.82707783688406	-2.18748548052271	-0.80272138781076
H	-0.83395172788909	-3.27918708034387	-0.65391251447171
H	-1.08132257777097	-1.97505326001348	-1.85615305410369
H	0.19878489709258	-1.81621012676695	-0.62845299377938
C	-1.96729051398686	-3.36909333282501	5.68459157943953
O	-1.63715151508474	-2.67834912055592	6.61555848008453
O	-2.46128160228938	-4.60215594138120	5.80683486302549
C	-2.63775258049740	-5.12278037274059	7.13140112844679
H	-1.67169559850080	-5.19194075422221	7.66092018389644
H	-3.33234828782554	-4.49335515058131	7.71456605773726
H	-3.06501715019091	-6.12950934306616	7.01105827343482
H	-3.12784335742985	2.11306137570536	1.39485998016562
H	-4.74706909138817	1.36301604744543	1.34723179344227
C	-1.01472057665654	-3.96474994907870	3.42554823176229
H	0.00630659485056	-3.98315041719228	3.85075690786820
H	-1.42894760681657	-4.98450468042509	3.48493323545858
H	-0.94495350234731	-3.67764841541980	2.36498059183923
Cl	-1.46359223444447	1.11487200752060	-0.76985584479040
CTA-g			
C	-3.11121562793883	-2.90844198633877	0.50216016978107
S	-4.78900312160787	-2.51779187941704	0.83803210503687
S	-2.37790513449870	-4.37738367886255	0.68302718691441
H	-4.55041616740652	-5.33583061798135	2.16107688703324
C	-5.55489152247871	-4.88550050196037	2.19617968191330
H	-6.29626010456203	-5.70240938372274	2.27685939401969
H	-5.62901763059922	-4.26184365595454	3.10240974861658
C	-5.82646462344885	-4.07151656997688	0.91679949647027
C	-7.26435826758270	-3.49794763443574	0.99830963766812
O	-8.16218772969217	-3.80885697253947	0.25609375011280
O	-7.39732058880050	-2.64003161752894	2.01020050325530
C	-8.68557506190245	-2.04387454831032	2.21436628348212
H	-8.99823501764906	-1.46324727789490	1.32902155895471
H	-8.57848211441361	-1.37046443490340	3.07782020015625
H	-9.44613073071269	-2.81239807668238	2.43662992875090
C	-5.69207193446804	-4.91070248391611	-0.35665219731846
H	-4.67487404021731	-5.32134415921308	-0.45122867192055
H	-6.39613888587249	-5.76018630191548	-0.31414329107420
H	-5.92124170262878	-4.32258398696999	-1.26138641265886
C	-2.36148072854958	-1.69589909871512	0.10185329183274
C	-2.93217468147547	-0.66794091217531	-0.68070786318922
C	-1.01117657786694	-1.53490996153853	0.48275669169955
C	-2.20489713768697	0.44893819036468	-1.06566362839336
C	-0.27546844259206	-0.41810440664931	0.12427349317786
C	-0.84853246360762	0.61886434494542	-0.66842715296419
H	-3.96642220320453	-0.75635803271439	-1.03457240458261
H	-0.53434573547557	-2.30808055415991	1.09732510214939
H	-2.69873470900325	1.19535340853524	-1.69506076798845
H	0.75973242346232	-0.34563851020336	0.47107156266763
N	-0.12880544654324	1.72100908662379	-1.03070853198446
C	-0.74625617704676	2.77163711746504	-1.82609517042519

H	-1.62429675381401	3.21536932422886	-1.31737809350792
H	-0.01643607727667	3.57782416349822	-1.99377683996626
H	-1.07522163834243	2.40533089777458	-2.81857122202601
C	1.26363409354820	1.85410588979266	-0.62852814855592
H	1.89038510710405	1.02936030833808	-1.02065422728859
H	1.66810299913446	2.79723123882215	-1.02549256496790
H	1.37901215571678	1.87761027429132	0.47292451511938

Et_2N-C(=S)-S-C(Me)(Et)CN S_0

N	-2.36653428679424	-1.42960655485163	1.12219907687882
C	-2.63829278085963	-1.47067560921872	2.44130179781383
S	-3.14083034610816	-0.14206774698227	3.32852415022967
S	-2.39635014206879	-3.10051355859204	3.14948316751035
C	-2.82298324696760	-3.06853736418819	4.97195251667233
C	-2.49956308986374	-4.47746285697469	5.30987381949124
N	-2.25712361946247	-5.57257176305978	5.59369094819596
C	-4.32817896114648	-2.85450440244568	5.22165655015143
H	-4.61733050420328	-1.83826870451388	4.91258734774342
H	-4.93638036637077	-3.58581581638355	4.66319552419535
H	-4.54286330082918	-2.96889626222172	6.29915971460866
C	-1.93793766614785	-2.14605036969527	5.85308148882126
H	-2.16795284284091	-2.42024913870325	6.90095339620609
H	-2.29613779069389	-1.11360103496794	5.71302964599234
C	-0.43261559413357	-2.22072809373760	5.61061137306879
H	0.09575115287686	-1.61066672178120	6.36416127154792
H	-0.04143285810692	-3.25220133358540	5.69148252374906
H	-0.15508136059961	-1.82845633035412	4.61598537254515
C	-2.47729373176422	-0.12983571454680	0.41690861750837
C	-2.58662166962344	-0.21469253188721	-1.10087147205212
H	-1.61417936835353	0.50355848633723	0.70614078746874
H	-3.37199002314804	0.37591380528541	0.81475006582761
H	-2.75885529585015	0.80773617610484	-1.48063924276375
H	-1.67200225264516	-0.59072154019507	-1.59119590374658
H	-3.44360597479748	-0.83273194977925	-1.42357697388912
C	-1.75353947232203	-2.55931581081645	0.39705582690494
C	-0.22545481631800	-2.53636941277158	0.43335019545058
H	-2.11346426926534	-2.53003453146235	-0.64254548139947
H	-2.14616857000271	-3.50763601048600	0.79984917056831
H	0.17298206244409	-3.40125077969888	-0.12575488223295
H	0.17754471405862	-1.61924634329810	-0.03237884625619
H	0.16011230190771	-2.59434507052878	1.46699009318984

References

[1] Wang, Z.; Wu, C.; Liu, W. NAC-TDDFT: Time-Dependent Density Functional Theory for Nonadiabatic Couplings. *Acc. Chem. Res.* **2021**, 54, 3288-3297.

[2] Li, Z.; Liu, W. Spin-adapted open-shell time-dependent density functional theory. III. An even better and simpler formulation. *J. Chem. Phys.* **2011**, 135, 194106.

[3] Zhang, Y.; Suo, B.; Wang, Z.; Zhang, N.; Li, Z.; Lei, Y.; Zou, W.; Gao, J.; Peng, D.; Pu, Z.; Xiao, Y.; Sun, Q.; Wang, F.; Ma, Y.; Wang, X.; Guo, Y.; Liu, W. BDF: A relativistic electronic structure program package. *J. Chem. Phys.* **2020**, 152, 064113.

[4] Niu, Y.; Li, W.; Peng, Q.; Geng, H.; Yi, Y.; Wang, L.; Nan, G.; Wang, D.; Shuai, Z. MOlecular MAterials Property Prediction Package (MOMAP) 1.0: a software package for predicting the luminescent properties and mobility of organic functional materials. *Mol. Phys.* **2018**, 116, 1078-1090.

[5] Neese, F. The ORCA program system. *WIREs Comput. Mol. Sci.* **2012**, 2, 73-78.

[6] Neese, F. Software update: the ORCA program system, version 4.0. *WIREs Comput. Mol. Sci.* **2018**, 8, e1327.

[7] Neese, F.; Wennmohs, F.; Becker, U.; Riplinger, C. The ORCA quantum chemistry program package. *J. Chem. Phys.* **2020**, 152, 224108-224108.

[8] Neese, F. Software update: The ORCA program system—Version 5.0. *WIREs Comput. Mol. Sci.* **2022**, 12, e1606.

[9] Neese, F. Software Update: The ORCA Program System—Version 6.0. *WIREs Comput. Mol. Sci.* **2025**, 15, e70019.

[10] Lin, Y.; Li, G.; Mao, S.; Chai, J. Long-range corrected hybrid density functionals with improved dispersion corrections. *J. Chem. Theory Comput.* **2013**, 9, 263-272.

[11] Weigend, F., Ahlrichs, R. Balanced Basis Sets of Split Valence, Triple Zeta Valence and Quadruple Zeta Valence Quality for H to Rn: Design and Assessment of Accuracy. *Phys. Chem. Chem. Phys.*, **2005**, 7, 3297-3305.

[12] Marenich, A. V.; Cramer, C. J.; Truhlar, D. G. Universal Solvation Model Based on Solute Electron Density and on a Continuum Model of the Solvent Defined by the Bulk Dielectric Constant and Atomic Surface Tensions. *J. Phys. Chem. B*, **2009**, 113, 6378.

[13] Casanova, D.; Krylov, A. I. Spin-Flip Methods in Quantum Chemistry. *Phys. Chem. Chem. Phys.* **2020**, 22, 4326-4342.

[14] Lian, S.; Armes, S. P.; An, Z. Universal Visible-Light Photoiniferter Polymerization. *CCS Chem.* **2024**, 7, 2304-2314.
